# Application of improved and efficient image repair algorithm in rock damage experimental research

**DOI:** 10.1038/s41598-024-65790-y

**Published:** 2024-06-27

**Authors:** Mingzhe Xu, Xianyin Qi, Diandong Geng

**Affiliations:** 1 Present Address: School of Urban Construction, Yangtze University, Jingzhou, 434023 China; 2 State Key Laboratory of Geomechanics and Geotechnical Engineering, Wuhan Institute of Rock and Soil Mechanics, Chinese Academy of Sciences, Wuhan, 430071 Hubei China

**Keywords:** Digital image, Image restoration, Transformer algorithm, Neural network, Rock damage, Engineering, Civil engineering

## Abstract

In the petroleum and coal industries, digital image technology and acoustic emission technology are employed to study rock properties, but both exhibit flaws during data processing. Digital image technology is vulnerable to interference from fractures and scaling, leading to potential loss of image data; while acoustic emission technology is not hindered by these issues, noise from rock destruction can interfere with the electrical signals, causing errors. The monitoring errors of these techniques can undermine the effectiveness of rock damage analysis. To address this issue, this paper focuses on the restoration of image data acquired through digital image technology, leveraging deep learning techniques, and using soft and hard rocks made of similar materials as research subjects, an improved Incremental Transformer image algorithm is employed to repair distorted or missing strain nephograms during uniaxial compression experiments. The concrete implementation entails using a comprehensive training set of strain nephograms derived from digital image technology, fabricating masks for absent image segments, and predicting strain nephograms with full strain detail. Additionally, we adopt deep separable convolutional networks to optimize the algorithm’s operational efficiency. Based on this, the analysis of rock damage is conducted using the repaired strain nephograms, achieving a closer correlation with the actual physical processes of rock damage compared to conventional digital image technology and acoustic emission techniques. The improved incremental Transformer algorithm presented in this paper will contribute to enhancing the efficiency of digital image technology in the realm of rock damage, saving time and money, and offering an innovative approach to traditional rock damage analysis.

## Introduction

In rock mining, when the rock mass is subjected to external forces, phenomena such as the development, penetration, and fragmentation of microcracks occur, which can easily lead to collapse and instability. To ensure mining safety and structural stability, it is essential to delve into the damage mechanisms of sedimentary rock layers. In this context, the capabilities of digital image correlation techniques (DIC), which offer dynamic monitoring of the global displacement and strain fields, and acoustic emission (AE) technology, are increasingly favored in the exploration of rock mass damage.

DIC is a widely used non-contact optical digital technology for measuring material deformation. It involves using high-resolution images of a specimen before and after deformation to calculate correlations and obtain displacement fields and strain fields on the specimen surface. DIC strain nephograms allow direct observation of the deformation behavior of material areas, providing a reliable reference for studying rock failure processes and crack development. DIC has yielded numerous theoretical and experimental findings on the mechanisms underlying rock damage and crack development. For example, Xing et al.have conducted studies in the field of crack propagation, utilizing DIC technology to monitor the full-field strain and strain rate fields during dynamic rock compression, revealing that the size of crack propagation is related to the duration of localized strain occurrence^1^. Tang et al. investigated the mechanical properties of rocks by analyzing the damage changes in Compression–Tension (C/T) cycle tests using 3D-DIC. They discussed the characteristics both inside and outside the local damage zone^2^. Additionally, Wang et al. delved into the structure and physical properties of composite rocks. They employed DIC technology to monitor the three-dimensional deformation information of heterogeneous materials under uniaxial compression. Their findings indicated that the damage difference increases with the material difference. DIC strain nephograms not only have application value in observing crack propagation but also play a crucial role in studying the constitutive models of rocks^3^. For instance, Song et al. utilized DIC technology in uniaxial compression tests to calculate damage coefficients based on strain deviations. They selected larger strain points to effectively reflect the evolution of full-field damage^4^. Similarly, Xu et al. conducted generalized stress relaxation experiments using DIC technology to analyze the evolution law of surface strain in rocks. They discovered that the difference in the evolution rate between axial and radial strain values during rheology is positively correlated with overall strain changes^5^. Furthermore, Niu et al. combined DIC and indirect tensile tests to analyze the stress–strain development of rocks under tensile action. They integrated fracture and statistical damage theories to establish a damage constitutive model and determined the stress intensity factor at the crack tip under tensile action^6^. According to the research findings, it has been observed that there are errors in the strain data obtained through DIC technology, resulting in partial deviations between the backtracking curve and the curve obtained by the test machine itself, the primary reasons for these defects are attributed to the heavy reliance of DIC technology on the camera’s frame rate and the integrity of the speckle pattern^7^. During the process of capturing and recording material deformation through the camera, high frame rate cameras are more capable of detecting subtle surface deformations, whereas low frame rate cameras may produce blurred images due to overlong exposure. In the initial stages of the experiment, when the deformation of the specimen is minor, the impact of the camera’s frame rate on the results of DIC strain nephograms is not significant. However, as the experiment progresses and the specimen experiences displacement, missing sections, and other phenomena due to load-induced damage, issues such as insufficient lighting, overexposure, and speckle drop-off may arise during the DIC camera’s capture process. This, in turn, affects the recognition and tracking of feature points, leading to significant errors in the output data. Some scholars have devoted themselves to optimizing experimental protocols to reduce errors^8,9^. For instance, in terms of adjusting lighting conditions, Badaloni et al. conducted a comprehensive analysis of the effects of illumination and noise on DIC imaging quality by continuously capturing 50 images using a Canon high-performance camera in an environment with fiber optic light sources. Their study revealed that uncertainties such as noise and lighting conditions can be minimized as much as possible by using high-performance light sources during experiments^8^. However, even with the best settings, image quality can still be affected, highlighting that every optical optimization method inevitably influences the inherent and unavoidable noise of digital cameras. Regarding hardware facilities, Rubino et al. attempted to address the issue of low frame rate cameras failing to capture rock fractures in a timely manner by combining high-speed cameras with DIC technology. The research findings demonstrated that high-speed camera technology can record images at high frame rates and low noise levels^9^. However, the high-speed cameras and high-performance light sources used in the aforementioned studies are several times more expensive than standard equipment, making them prohibitively costly. Most small to medium-sized laboratories are unable to afford such high-end equipment due to limited experimental conditions and financial constraints. Additionally, even with the replacement of high frame rate cameras and matching camera shutters and lenses, errors caused by equipment are still difficult to avoid. Although optimizing experimental protocols helps reduce errors, it cannot eliminate the problem of errors. Furthermore, in the process of DIC data processing, the image undergoes dimension reduction, which leads to the loss of some detailed information or is affected by noise, resulting in a missing output image and affecting the accuracy of experimental results.

AE technology is frequently employed alongside DIC technology to monitor the failure process of rocks. AE technology detects AE phenomena by sensing transient pulse signals and determining the presence of stress wave signals. Each AE signal carries information about internal structure defects and the state of the rock mass, which can be analyzed to determine the displacement field and strain of the rock surface. In comparison to DIC technology, many experiments opt for AE alone or a combination of both technologies. For example, Du et al. conducted a series of experiments including Brazilian indirect tensile tests, three-point bending tests, and uniaxial compression tests to study the types of microcracks generated during the rock fracturing process^10^. By analyzing AE characteristics, they were able to determine the microcrack types and reveal the fracture mode of rocks, as well as the properties of the microcracks. Another study by Dai et al.combined DIC and AE technologies to quantitatively investigate the characteristics of rock fracture process zones. They were able to obtain information such as the locations of crack tips, crack extension lengths, and stress intensity factors through displacement fields and AE signals^11^. Li et al. used AE and DIC technologies to examine the effects of the spatial distribution of voids and joint combinations on the instability phenomenon of rock masses. The DIC results showed that changes in joint angles affected displacement fields and crack types, while the AE results indicated that shear cracks played a dominant role in the ultimate failure of specimens^12^. Gu et al. utilized AE technology to explore the effects of crack closure behavior on rock deformation. They derived damage evolution equations for four brittle rocks using AE ring counts^13^. L.M. studied material crack development and evolution using AE technology. They treated AE ring counts as characteristic parameters and proposed a damage constitutive model based on AE ring counts and strain^14^. When comparing damage strain curves constructed using AE and DIC technologies, it is evident that AE technology can compensate for the impact of missing data in DIC technology on experimental results, thereby providing theoretical references for DIC missing data. However, there are deviations between the curves where DIC data is not missing, indicating that AE technology also encounters issues of data loss or alteration in practical applications. AE technology effectively avoids problems related to missing images, but it can still be influenced by environmental noise, as well as pressure and temperature changes during experiments, resulting in data discrepancies. AE signals are generally weak and vulnerable to external noise, and the substantial noise generated by rock failure and machine operation can easily overshadow AE signals, thus affecting the accuracy of experimental results.

In recent years, with the rise of Artificial intelligence (AI) methods, although the issue of experimental noise is difficult to handle, AI image recognition and restoration provide new ways for improving DIC technology. Currently, AI methods are predominantly used for identifying and predicting rock cracks in various studies. For instance, Sidorenko et al. employed deep convolutional neural networks (CNN) to preprocess noisy Computed Tomography (CT) images and establish high-quality three-dimensional rock models^15^. Li et al. utilized multi-pixel point cloud coordinate regional growth segmentation models to accurately segment deformation areas of complex rock slopes under different rainfall conditions and prevent slope instability^16^. Tariq et al. combined support vector machines (SVM), artificial neural networks (ANN), and adaptive neural fuzzy inference systems (ANFIS) to predict crack growth and mitigate issues such as wellbore instability^17^. Similarly, Robson et al. employed convolutional neural networks to identify images and subsequently utilized object-based image analysis (OBIA) to classify the images, enabling the study of spectral similarity between rocks and surrounding materials^18^. These studies primarily focus on identifying and predicting damaged images during the rock failure process. While they leverage the advantages of AI methods in image processing, their treatment of damaged images is limited to clarity and target segmentation. Therefore, further exploration of the constitutive relationship of rocks is required. The study of rock damage evolution necessitates the utilization of DIC technology. Thus, addressing the existing gaps in DIC images through efficient restoration algorithms is crucial to enhancing the integrity and accuracy of DIC images. This will provide more reliable research data’s for investigating the process of rock damage evolution.

This article focuses on conducting uniaxial compression tests on soft and hard rocks using DIC and AE technology. The aim is to explore the limitations of these two technologies in the process of rock damage evolution. To address the issue of missing strain and displacement fields caused by speckle shedding in DIC technology, an incremental Transformer algorithm (ZITS) based on the Transformer is used to repair the speckle shedding areas in DIC images. After repair, the damaged areas with complete strain field information are extracted, thereby solving the consistency issues between DIC technology and AE technology in the field of rock damage.

## Compression test based on DIC and AE

### Test preparation and process

Due to the significant challenges in extracting cores from native rock, this experiment selected mudstone and sandstone from the dense composite strata of the Jimsar Basin as prototypes^19–22^. Utilizing principles of similarity theory, we fabricated artificial rock-like materials with physical and mechanical properties adhering to similarity laws^23^. The relationship between the original rocks and the model specimens is as follows:

1 $$C_{\sigma } = C_{\gamma } \cdot C_{L}$$

where *C*_*σ*_, *C*_*γ*_, *C*_*L*_ respectively represent the Stress ratio, Bulk density ratio, Geometric similarity ratio.

**Figure 1:**
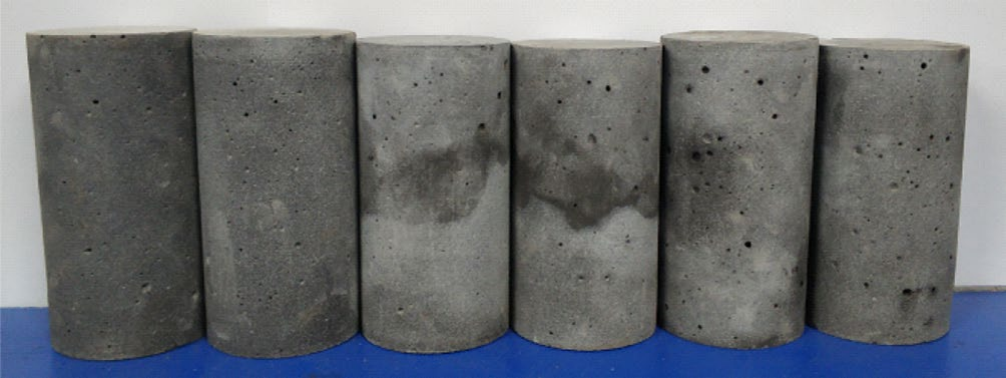
50 mm × 100 mm cylindrical rock specimens.

Core specimens were drilled using equipment into standard cylindrical rock specimens measuring 50 mm × 100 mm, as depicted in Fig. 1. The material mixture comprised cement, quartz sand, silica fumes, water, water-reducing agents, and defoamers, with a similarity ratio of cement:sand:water:defoamer = 1:0.7:0.35:0.003 to simulate the characteristics of soft rock (mudstone). The use of high-purity silica powder combined with a water-reducing agent enhances the strength and density of the rocks. To simulate the characteristics of hard rock (sandstone)^24^, a similar ratio of cement:sand:silica fumes:water:water-reducing agent:defoamer = 1:0.8:0.1:0.3:0.003:0.003 was employed. Experiments conducted with these parameters yielded two types of artificial rocks whose mechanical properties, including elastic modulus, are presented in Table 1.

**Table 1:** Similar material ratio and mechanical properties parameters.

Rock type	Uniaxial compressive strength (MPa)	Young’s modulus (GPa)	Mass ratio of cement, sand, silica powder, water, water reducer and defoamer	Stress ratio (*C*_*σ*_)	Bulk density ratio (*C*_*γ*_)	Geometric similarity ratio (*C*_*L*_)
Sandstone	178.86	26.21	–	3.73	1.2	3.11
Hard rock	47.9	6.84	1:0.7:0:0.35:0:0.003			
Mudstone	143.46	18.86	–	4.43	1.2	3.69
Soft rock	32.35	3.91	1:0.8:0.1:0.3:0.003:0.003			

In this experiment, a total of five groups of specimens were prepared, with each group consisting of 4 to 5 specimens each of the artificial soft rock and artificial hard rock, amounting to 47 specimens in total. The relevant physical indicators of the specimens were averaged. Following experimental testing and research analysis, the results from the 5 sets of specimens exhibited similar trends.

For the upcoming deep learning algorithm experiment discussed below, a sufficient number of images are required as the foundation of the dataset. Thus, this paper selects the images from the first four groups of specimens as the source for the training set of the dataset, while the images from the fifth group of specimens serve as the source for the validation set^25^. The first four groups of experiments encompass different strain distributions and variation patterns under various conditions, providing rich data foundations for the algorithm. The aim is to further enhance the accuracy and precision of the algorithm in strain measurement and repair through deep learning algorithm training. Simultaneously, the fifth group of specimens is utilized as the validation set to evaluate the effectiveness and generalization capability of the algorithm based on its performance. This ensures that the deep learning algorithm can consistently perform effectively under different conditions. Due to space constraints, this paper analyzes representative specimens from the fifth group, including specimens resembling soft and hard rocks.

**Figure 2:**
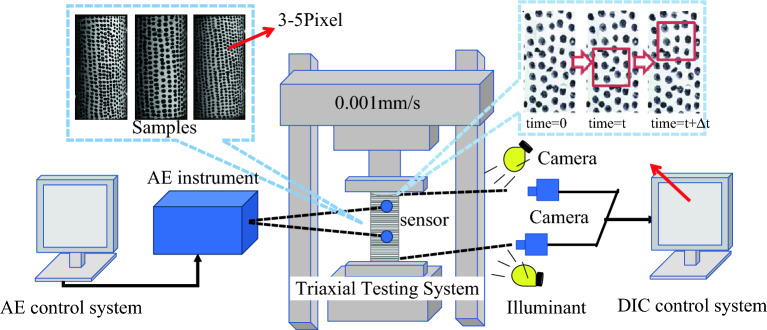
Diagram of uniaxial compression test system of rock acting together with 3D-DIC and AE.

The experiment was conducted using the HYAS-1000C rock triaxial testing system with a loading rate of 0.001 mm/s. The testing system is equipped with DIC and AE modules. The DIC hardware includes two cameras with resolutions of 2448 × 2048 pixels and a frame rate of 70 fps, along with lenses of 8 mm, 16 mm, 25 mm, and 35 mm, as depicted in Fig. 2. This setup enables the simultaneous acquisition of DIC dynamic strain data and AE data during uniaxial testing. The experimental procedure is as follows:

1. Random speckles were created on the surface of the rock specimens using spray paint, based on the resolution and measurement area of the DIC cameras.
2. The DIC and AE equipment are cleaned, and the camera undergoes a pre-heating check to ensure it maintains a stable frame rate when in operation.
3. Before the start of the experiment, the DIC and AE devices are assembled and connected to the system. The DIC measurement head is initialized, and the measurement distance and camera spacing are determined based on the lighting conditions. The cameras are calibrated using a calibration plate. Additionally, an ultrasonic transmitter and receiver are installed above and below the rock specimen surface to ensure full contact with the specimen.
4. The experimental parameters for AE testing were adjusted according to the current noise level measurement. The DIC acquisition frequency and step size best suited for image acquisition were determined through preliminary tests.
5. As stress loading commences, AE and DIC testing begin simultaneously. The camera captures images of the rock specimen at a set frequency, with an interval of 1 s per image, while the AE system continuously collects and analyzes the AE signals in real time.
6. After the experiment concludes, the collected AE data and DIC data are organized and analyzed to determine the deformation behavior of the rock specimen throughout the loading process.

**Figure 3:**
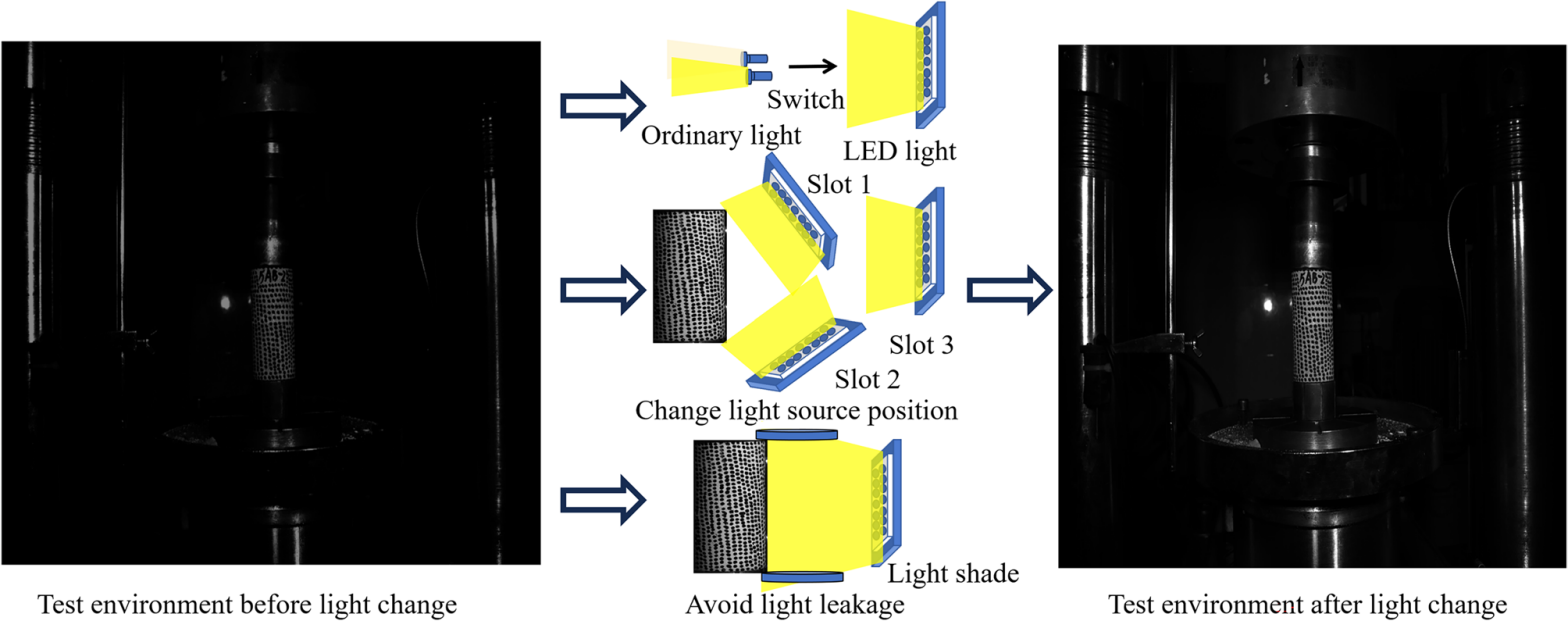
Before and after light conditions.

**Figure 4:**
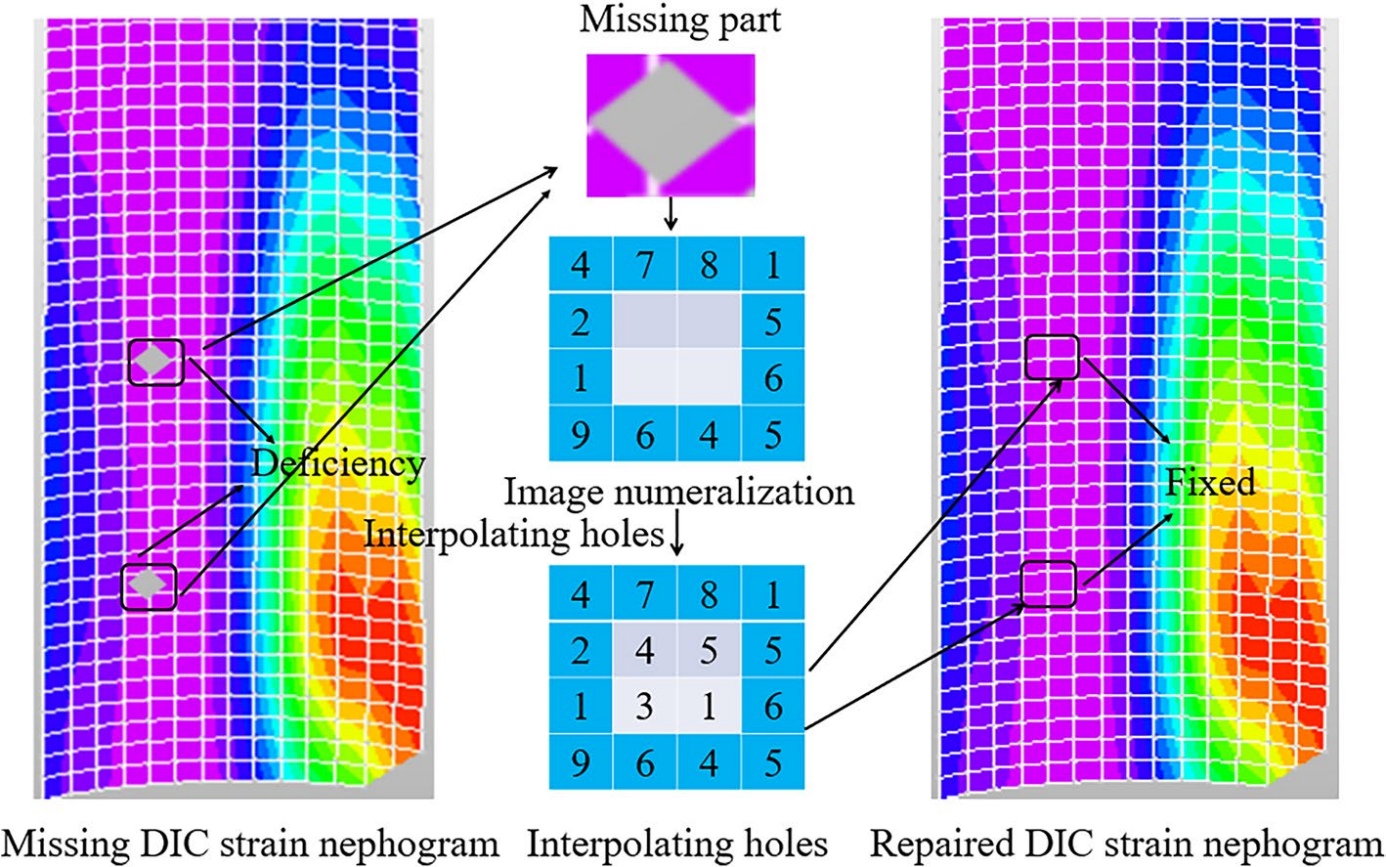
Interpolation and hole filling reconstruction before and after comparison.

Some scholars have conducted research on reducing errors inherent in experiments^8,9^, focusing on aspects such as lighting, camera shutter, and lens processing. Drawing upon the findings of these scholars, this paper employs methods of optimizing experimental settings before experimentation to mitigate these issues to some extent. The experiment at hand is optimized through the following approaches:

1. Adjusting lighting conditions. LED lights are selected as the light source, and their position and direction are adjusted by moving the light source or using brackets to ensure uniform illumination on the specimen. Additionally, light propagation is controlled using a light shield to prevent light leakage. Through pre-experimentation to compare optimal lighting conditions, ensuring images do not suffer from resolution differences or occlusions. A comparison of images before and after the change in lighting conditions is shown in Fig. 3 Before and after light conditions
2. Speckle creation. During speckle creation, contrast is adjusted to approximately 50% based on the most suitable lighting conditions, and particle size is controlled between 3–6 pixels to ensure accurate recognition of speckles by the camera. To capture DIC strain nephograms more effectively, the camera shutter speed is set to a maximum of 200 ms, based on the optimal speckle size of 3–5 pixels for the DIC system and the laboratory environment, to avoid issues such as image blurring due to overexposure or incomplete exposure.
3. Peripheral assistance. To ensure accurate capture of strain information before and after specimen failure by the DIC camera, a phase-locked loop system is utilized to adjust the DIC camera. Since the DIC camera lacks high-frequency shooting capabilities, the phase-locked loop system is employed for signal frequency division and delay processing, achieving stable high-frequency measurements by low-frequency devices.
4. Speckle patch reconstruction. Speckle information is systematically outputted as DIC strain nephograms. Due to various reasons, data loss or invalid areas may result in voids in the DIC strain nephograms. Therefore, this paper employs interpolation to fill voids as an experiment optimization technique. Interpolation is a post-processing step that uses functions to calculate neighboring pixel values and fill missing information. A comparison of the reconstruction before and after is shown in Fig. 4. Interpolation and hole filling reconstruction before and after comparison

### Rock damage analysis based on compression test

#### Analysis of AE ringing count-time curve

**Figure 5:**
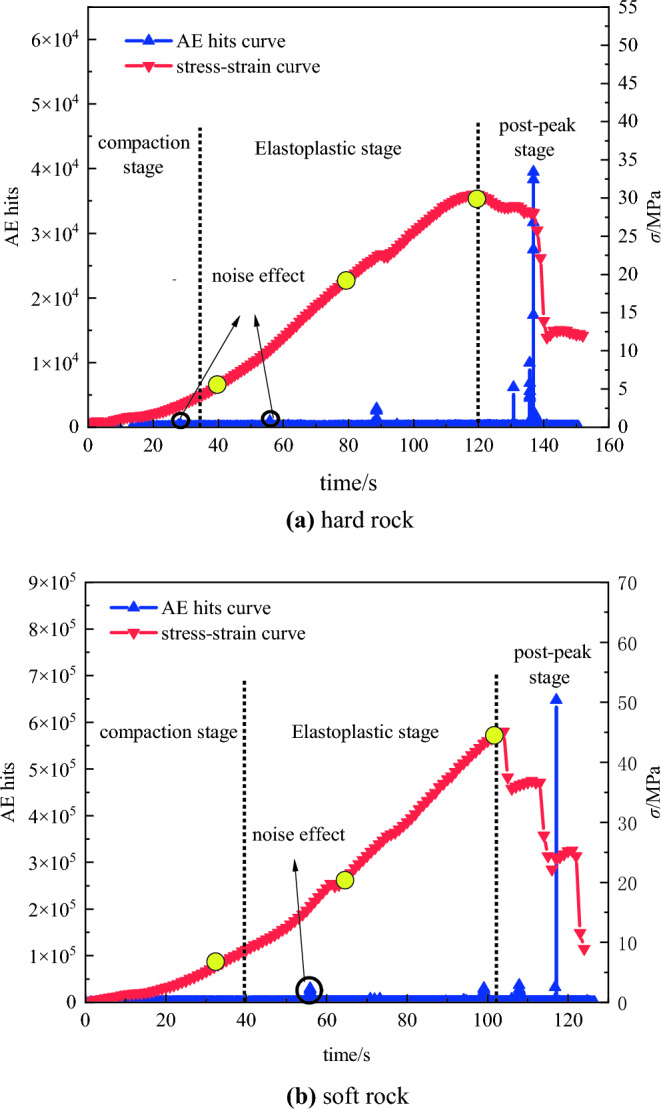
The variations of AE hit and loads versus time in different rock specimens: (**a**) hard rock, (**b**) soft rock.

The AE technique monitors the deformation process of rocks during uniaxial compression by measuring the number of electrical signals exceeding a threshold, known as AE events^26^. In Fig. 5, the graph illustrates the changes in AE counts and stress over time for both hard rock and soft rock specimens.

As shown in Fig. 5, the overall curve for hard rock and soft rock specimens exhibits similar changes. Initially, during the compaction and elastic stages, hard rocks, with their lower porosity and incomplete closure of internal pores, experience greater local deformation and damage accumulation compared to soft rock specimens. Consequently, hard rocks exhibit a higher frequency of AE events, resulting in small fluctuations in the ring-down count curve. As both specimens enter the plastic stage, surface cracks begin to form, leading to a slight increase in the AE ring-down count. During the post-peak failure stage, cracks in hard rock specimens expand and penetrate, causing extensive detachment of the specimen surface and a significant increase in AE events. The ring-down count curve reaches its peak when the specimen is destroyed, followed by a gradual decrease and convergence to a straight line. In contrast, soft rock specimens only exhibit one distinct ring-down peak during the complete failure stage.

After conducting extensive research, scholars have discovered a strong correlation between the AE ring-down count and the progression of internal structural defects in rocks. L.M proposed a damage index defined by the change in ring-down count in cross Sections^14^, as follows:

2 $$D_{AE} = \frac{{N_{d} }}{{N_{0} }}$$

where *N*_*d*_ is the cumulative AE ring-down count when micro-defects in the bearing section reach a certain level, where *N*_*0*_ is the cumulative AE ring-down count when the bearing section is completely destroyed.

**Figure 6:**
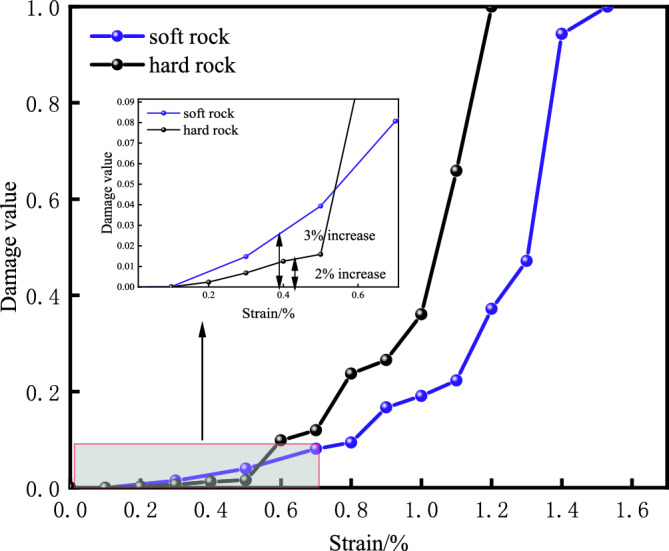
AE damage-strain curves of two specimens.

As shown in Fig. 6, during the initial compaction stage, the damage progression in both types of rocks is relatively gradual. In the plastic phase, which corresponds to the development period of microcracks, the curve of rock damage accelerates from a slow to a fast rise. In the post-peak failure stage, the damage values quickly escalate to reach 1. However, during the actual compression process of the rocks, there is virtually no change in damage during the initial compaction, but the rock damage estimated based on AE statistics may show an increase of 2 to 3% over the actual damage values due to the inherent noise from the operation of the testing machine. Additionally, during the plastic and post-peak failure stages, factors such as extensive specimen damage, intense noise from failure, and sensor movement can contribute to an increased ringing count. Consequently, AE-based statistics may overestimate rock damage values compared to the actual values.

#### Damage analysis based on DIC strain nephogram

**Figure 7:**
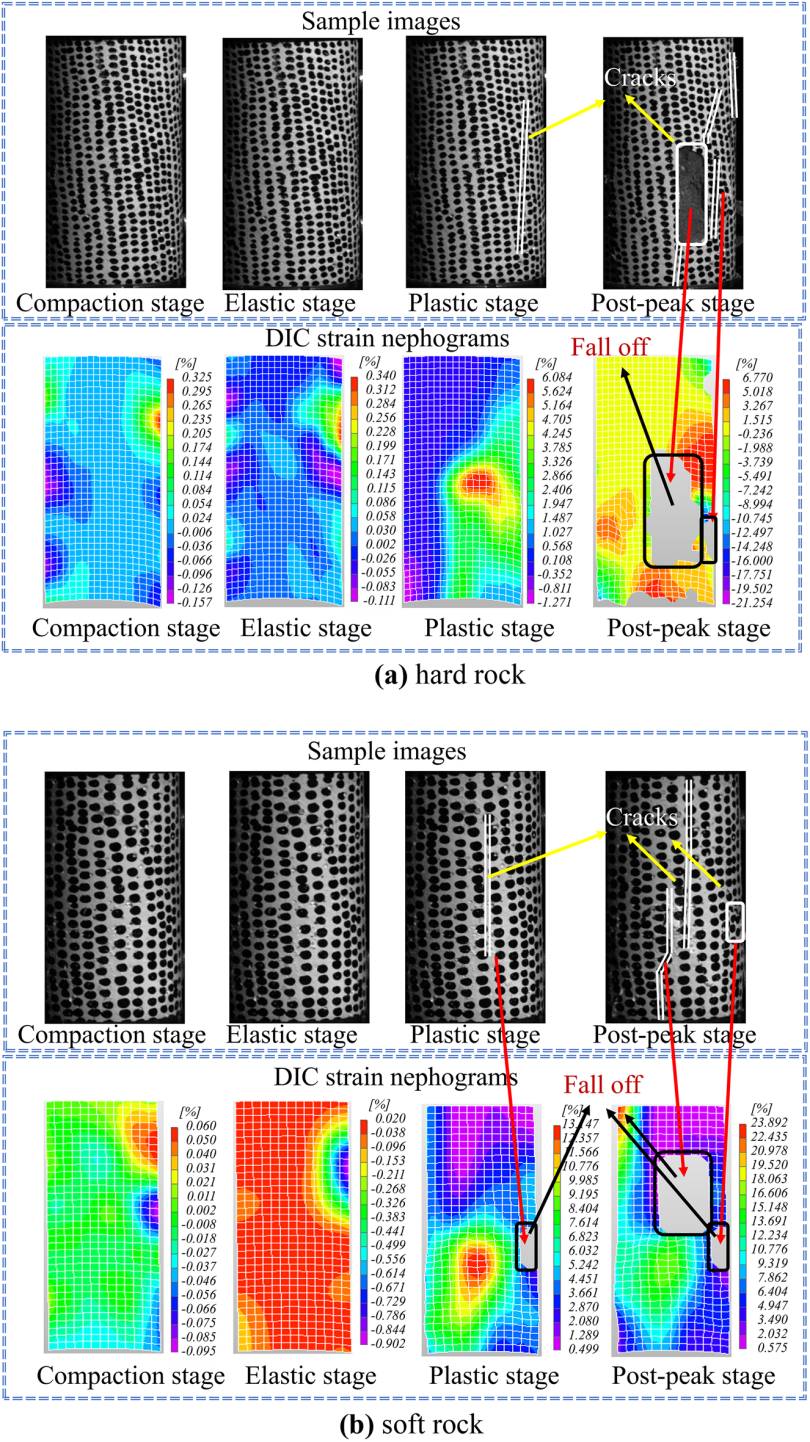
Failure process diagram of different rock specimens and corresponding DIC strain nephogram: (**a**) hard rock, (**b**) soft rock.

The DIC strain nephogram offers comprehensive strain distributions across the specimen surface by sampling points^27^. By comparing and analyzing the DIC strain nephogram at the intersection points of different loading stages, it becomes possible to effectively observe and understand the damage and failure characteristics of the specimen. Figure 7 showcases the rock failure images and instantaneous DIC strain nephograms for both hard rock and soft rock specimens under uniaxial compression at various loading stages.

According to Fig. 7a, During the initial compaction stage, the strain distribution is uniform, and the species remains relatively unchanged, the local maximum strain is only 0.325%. Subsequently, as the hard rock underwent densification and elastic stages, its high compressive strength and load-bearing capacity led to minimal surface alterations^28^, with only a slight amount of strain visible on the strain nephogram. With increasing load intensity, small cracks started appearing on the specimen surface during the plastic deformation stage^29,30^, gradually expanding until they penetrated the specimen from top to bottom, resulting in fragment spalling. At this juncture, the local maximum strain peaked at 6.084%. Once the peak strength is reached, the stress–strain curve drops rapidly, and the specimen enters the failure stage. The cracks within the specimen develop quickly and intersect to create a visible macroscopic fracture surface. In various force areas of the strain nephogram, distinct boundaries can be observed, highlighting the obvious stress concentration characteristics with a maximum local strain of 6.770%. Ultimately, the specimen underwent complete failure, forming macroscopic tensile cracks.

In Fig. 7b, the strain trend of soft rocks is similar to that of hard rocks. However, the failure process of the hard rock curve is longer during the post-peak failure stage, indicating certain ductile characteristics. Based on the strain nephogram, the local maximum strain on the surface of the soft rock is 13.147%, which is 7.063% higher than that of the hard rock, indicating that soft rock has poor compressive strength. When the deformation on the rock surface is small, the rock specimen has already reached the ultimate compressive strength and failure occurs. The specimen mainly fails in shear, with some local tensile failure^31,32^.

**Figure 8:**
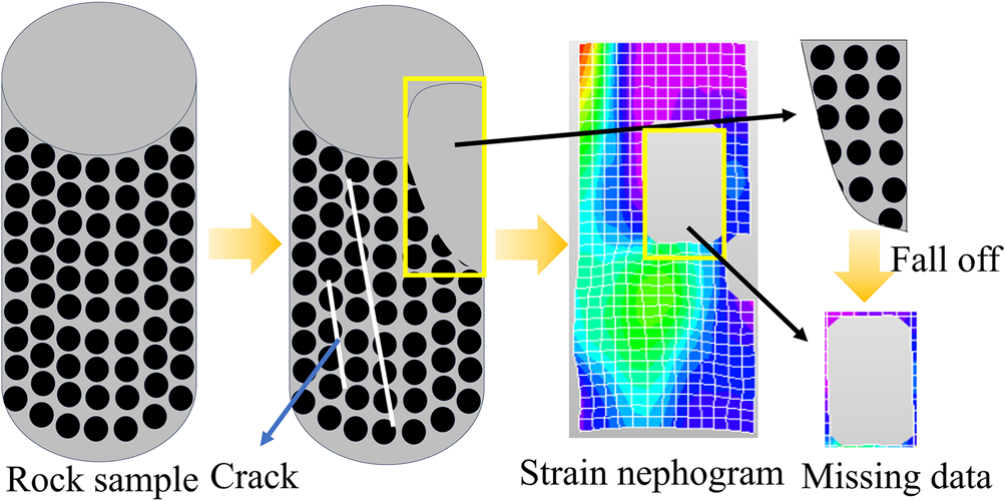
The process diagram of speckle shedding when rock specimen is damaged.

**Figure 9:**
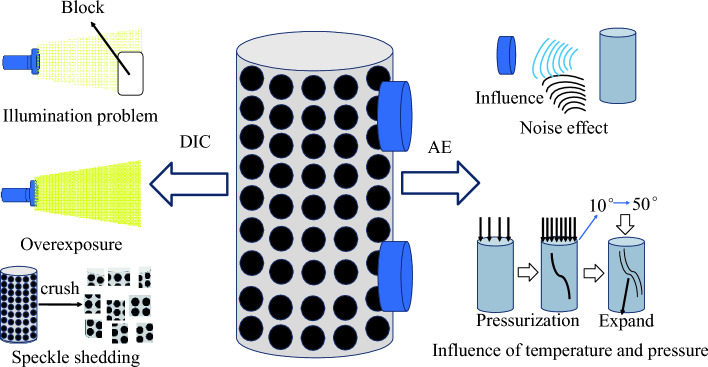
Defect diagram of DIC and AE system in uniaxial compression test of rock.

The comprehensive analysis indicates that while DIC strain nephograms can somewhat intuitively reflect the process of rock specimens under loading, from the initiation and expansion of local cracks to their coalescence, there are inevitable shortcomings when using DIC strain nephograms to detect the progression from initial loading to failure in rocks, due to the nature of their measurement. DIC strain nephograms are generated by tracking the deformation of speckle patterns on the object’s surface and calculating changes in the grayscale values of speckle domains, to obtain the deformation and strain data of the tested surface. Consequently, their use in rock diagnostics during the entire loading to failure process can result in the following deficiencies: (1) Blurred boundaries: As shown in Fig. 7, when the rock is in the initial compaction or elastic phase, the deformation and strain produced by the rock surface are inherently small. When influenced by objective factors such as ambient lighting, camera resolution, and speckle domain deviations^33^, the strain nephograms derived from DIC algorithms are prone to blurred boundaries and mixed areas. (2) Loss of information: As shown in Figs. 8 and 9, the formation of DIC strain nephograms heavily depends on the speckle patterns on the rock surface^34^. The loading forces can create various cracks and even cause delamination of the surface, thereby damaging the speckle domains and leading to loss of information from these regions. Therefore, to ensure the completeness of the detection results and the accuracy of the observed strain patterns, the DIC technique for rock diagnostics still requires further refinement.

Typically, the DIC technique is employed to analyze the quantitative relationship between damage and strain in rocks during uniaxial compression tests. Essentially, this involves using DIC technology to acquire global strain data of the rock throughout the loading to failure process. Subsequently, this data is converted through the relationship between damage and strain to calculate the final damage curve of the rock. Song identified in uniaxial rock experiments that the initial 5 to 10% of high-strain points are closely related to the evolution of damage concentration^4^. They also found that the average value of all strain points reflects that most measurement points are in a state of uniform deformation. Based on these findings, it is proposed to evaluate the extent of specimen damage by the difference in the average values of these two metrics, denoted as $\overline{\varepsilon }$, that is to say:

3 $$\overline{\varepsilon } = \frac{1}{M}\sum\limits_{i = 1}^{M} {\left( {\varepsilon_{x} } \right)_{i} } - \frac{1}{N}\sum\limits_{i = 1}^{N} {\left( {\varepsilon_{x} } \right)_{i} }$$

where $\frac{1}{M}\sum\limits_{i = 1}^{M} {\left( {\varepsilon_{x} } \right)_{i} }$ is the average value of the first *M* larger strain points, where $\frac{1}{N}\sum\limits_{i = 1}^{N} {\left( {\varepsilon_{x} } \right)_{i} }$ is the average value of strains across all measuring points.

On this basis, the damage severity factor *D* was defined as follows:

4 $$D = \frac{{\overline{\varepsilon }}}{{\overline{\varepsilon }_{\max } }}$$

where $\overline{\varepsilon }_{\max }$ is the maximum value of *ε*, which is the critical failure value.

**Figure 10:**
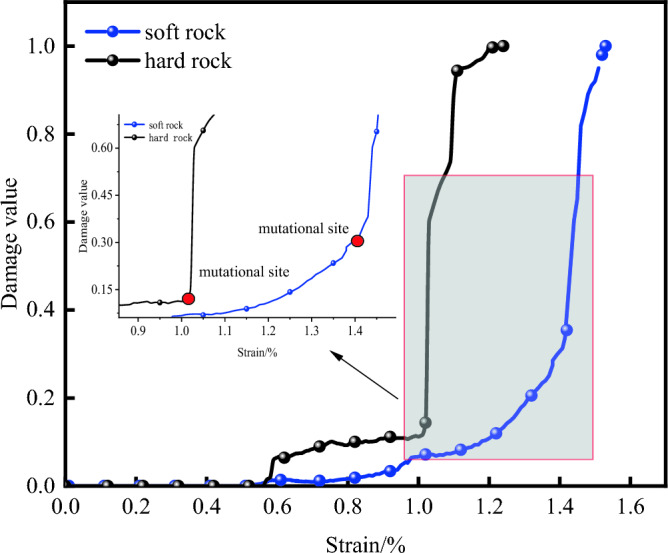
DIC damage-strain curves of different rock specimens.

As shown in Fig. 10, damage mutation points appear at 1.0% strain for hard rocks and at 1.4% strain for soft rocks, after which the damage value rapidly rises to 1. This occurrence is because although DIC strain nephograms allow for the observation of the dynamic failure process of the specimens, actual specimen failure can lead to speckle swelling or detachment, resulting in the loss or inaccuracy of some strain data. Consequently, the corresponding global strain averages obtained through DIC technology decrease, resulting in damage values on the curve being smaller than the actual compression process of the rock. Therefore, obtaining valid full-field strain information across the entire compression process of rock is one of the key technical challenges to be addressed in the subsequent sections of this study.

### Difference analysis of rock damage curves between DIC and AE techniques

**Figure 11:**
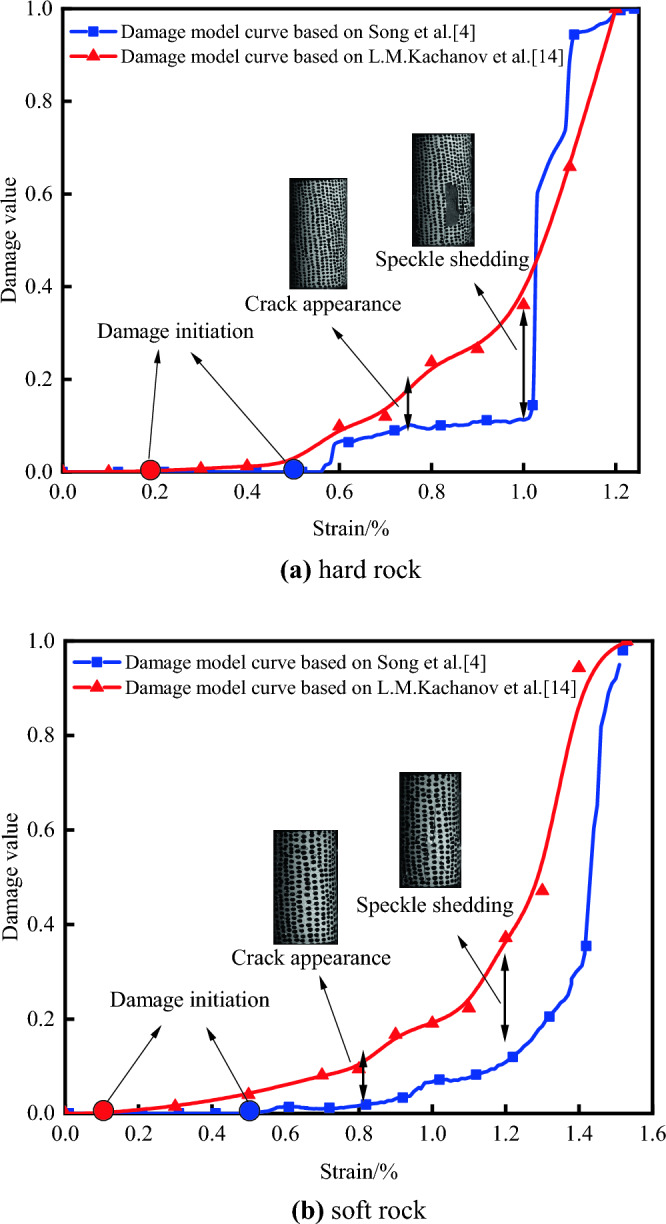
Comparison of damage-strain curves in different rock specimens based on DIC and AE techniques: (**a**) hard rock, (**b**) soft rock.

As depicted in Fig. 11, notable differences exist between two monitoring technologies, DIC and AE, in the analysis of rock damage. Firstly, there is a discrepancy in the onset point of damage between the two curves. In actual conditions, rocks generally show no damage variation during the initial consolidation and elastic stages. However, as Fig. 11 suggests, the AE-based damage curve indicates some damage occurring at this stage. This could be due to the influence of noise generated by the testing machine during the monitoring of AE signals, leading to irrelevant signals passing through the AE counting threshold, thereby causing monitoring errors and resulting in damage generation during curve fitting. On the other hand, the DIC-obtained curve shows damage onset occurring much later during or after the plastic phase transition. This delay is attributed to the DIC software losing edge data through dimension reduction processing, shifting the starting point of damage. Secondly, there are distinct differences in the transition trends of the two curves as they enter the plastic phase and post-peak failure stages. In the damage analysis based on DIC technology, when the specimen enters the plastic phase under uniaxial compression conditions, the initiation of microcracks and the subsequent expansion of surface cracks cause speckle division. When DIC technology maps the strains from the speckles, the cracked areas appear as voids in the strain nephograms, resulting in data loss and an inability for the DIC damage curve to correctly reflect the inflection at this phase. In contrast, with the AE-based damage analysis, when specimens suffer a breakage due to stress during this phase, the noise generated interferes with the propagation of AE signals, leading to a significant deviation between the AE and DIC damage curves. In the post-peak failure phase, the rock suffers extensive damage, with a portion of the speckles peeling off with the outer surface, resulting in widespread data loss and a severely affected strain dataset. This causes the DIC-based damage curve to ascend rapidly during this stage. Meanwhile, the AE-based damage curve is prone to misjudgments under high-intensity environmental noise, mistaking noise for AE signals and thus increasing ringing counts. As a result, the damage values obtained from AE measurements are higher than the actual damage values of the rocks.

**Figure 12:**
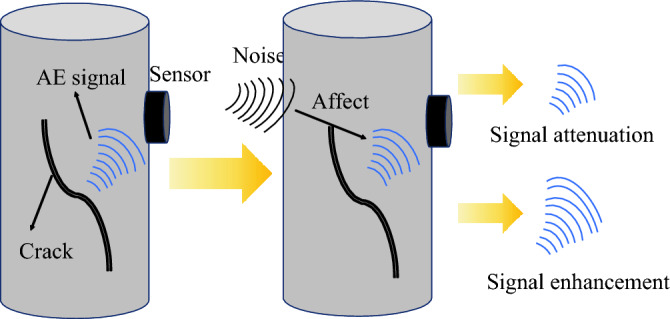
The process diagram of acoustic emission signal changes under the influence of noise.

In summary, it can be concluded that damage analysis based on AE technology relies heavily on precise ringing count measurements, while the analysis based on DIC technology depends on the full-process speckle pattern strain nephogram. During the uniaxial compression test of rocks, AE technology can detect the initiation and propagation of microcracks in real-time. However, the operational noise from the testing machine can either amplify or diminish the AE signals, as shown in Fig. 12. AE ringing count refers to the number of oscillations after the waveform exceeds a threshold voltage, and high-intensity environmental noise can lead to false counts from irrelevant signals, diminishing the precision of the data. Furthermore, during the experimental process, the detachment of AE sensors can also result in data anomalies. The sensors, which are attached to the surface of the specimen to capture AE signals and convert them into electrical signals for software analysis, can detach, shift, or be obstructed during rock failure, leading to challenges in signal collection and localization, and thus affecting the assessment of damage severity.

The main issue with DIC technology in rock compression damage analysis is its high reliance on the full-process coordinate changes of speckle points; the loss of these points leads to missing global strain field information, also negatively impacting damage analysis. Current solutions to address noise interference in AE technology include using sensors with high sensitivity and low noise characteristics, positioning sensors away from noise sources with appropriate shielding measures, and employing filtering and noise reduction algorithms during signal processing to extract useful AE signals and suppress noise. Sensor improvement plays a significant role in noise reduction but is hindered by the high cost of precision sensors, which limits further development of AE technology. Meanwhile, faced with the issue of missing data in DIC speckle strain nephograms, numerous scholars have used artificial intelligence algorithms to infer missing area information through the diffusion of boundary information or generate pixel blocks based on existing image information to supplement the image data^35–37^. Compared to the sensor improvement approach in AE, the DIC image enhancement techniques based on algorithmic methods offer a better cost–benefit ratio. Therefore, this article addresses the issue of damage analysis errors arising from both monitoring technologies by employing image restoration methods on DIC strain nephograms. Herein, a Transformer-based ZITS algorithm is proposed to repair and predict missing speckle areas in DIC strain nephograms, the objective is to recover the lost strain information of monitoring points, segment, and distinguish between different strains. On this basis, by recognizing the damaged pixels in the repaired DIC images as areas of rock damage and utilizing a new damage quantification model, the study compares it against two traditional damage models to assess the impact of data loss issues on research outcomes.

## An improved image crack recognition method based on deep learning

Before deep learning became prevalent, image restoration relied on traditional learning algorithms, such as patch-based restoration algorithms and pixel diffusion-based image restoration algorithms. These methods suffered from low task accuracy and poor handling of detail and texture^38,39^. With the rise of deep learning, a wealth of algorithmic research has provided opportunities for breakthroughs in the challenging field of image restoration. Numerous scholars have found that CNN and autoencoders hold unique advantages in image processing. Among them^40–42^, the U-Net algorithm, which is based on a CNN architecture, and the Transformer algorithm, rooted in an autoencoder structure, are currently the most widely used models for image processing tasks. Chapter 3.1 will analyze and compare the strengths and weaknesses of these two image restoration algorithms.

### Comparison of common repair algorithms

**Figure 13:**
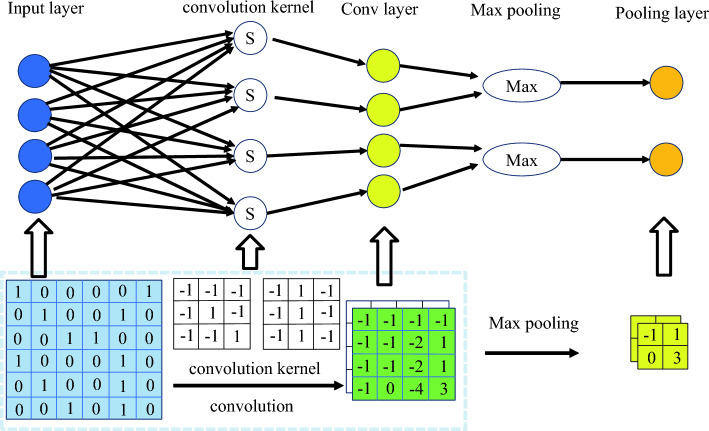
Convolution with Max pooling graph.

The U-Net algorithm is based on convolutional neural networks, which consist of convolutional layers and pooling layers^43^, as shown in Fig. 13. Convolutional layers process image pixels into feature values, which are then classified into corresponding features through matrix transformations. The pooling layer performs screening by selecting important feature values from the output of the convolutional layer, resulting in the output of a target feature image. The U-Net algorithm primarily utilizes pixel reconstruction for image restoration, offering benefits such as image invariance, image classification, and segmentation. However, the U-Net algorithm is time-consuming in image processing and requires a large dataset for label creation. The quality of experimental results is heavily reliant on the dataset used. Moreover, the restoration outcomes are significantly impacted by the receptive field, which denotes the area in the input image that each layer’s feature map corresponds to during pixel mapping. Consequently, the size of the convolutional layer limits the receptive field, and the restored image may not accurately reflect the output features.

**Figure 14:**
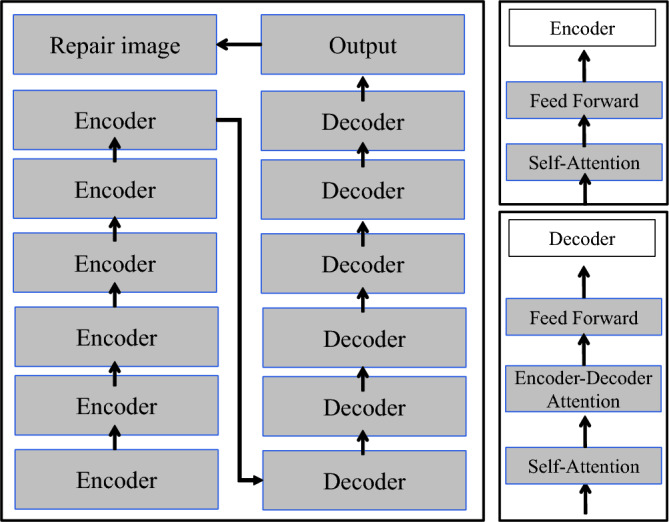
Transformer structure diagram.

The Transformer algorithm consists of a left half with six layers of encoders and a right half with six layers of decoders. As shown in Fig. 14, the encoder is responsible for extracting features, while the decoder is responsible for restoring the image information^44^. After these two stages, the data is classified using the *Softmax* function to generate the final restoration result. The *Softmax* function is defined by the equation provided below:

5 $$Softmax\left( {Z_{i} } \right) = \frac{{{\text{e}}^{Zi} }}{{\sum\nolimits_{j = 1}^{K} {{\text{e}}^{{Z_{j} }} } }}$$

where *Z*_*i*_ is the input vector, where *K* is the number of classifier classes.

The image information is processed through the encoder and decoder, where the calculation part mainly consists of two components: multi-head attention layer and feed-forward neural network. These two components are connected end-to-end, with the feature processing starting with the multi-head attention layer. The multi-head attention layer is composed of multiple self-attention mechanisms. The self-attention mechanism involves three weight matrices: *Q* (query vector), *K* (key vector), and *V* (value vector), which are used to pre-calculate the attention weights at each position for the input data. Subsequently, the weighted sum is computed to generate features at different levels. The formula for the self-attention mechanism is as follows:

6 $$Attention\left( {Q,K,V} \right) = softmax\left( {\frac{{QK^{T} }}{{\sqrt {d_{k} } }}} \right)V$$

where *d*_*k*_ is the column size of the matrices *Q* and *K*, which corresponds to the vector dimension.

Feed-forward neural networks are a type of one-way, multi-layer network structure, where each layer of neurons can only receive signals from the previous layer and output to the next layer. However, the output of the feature by the multi-head attention layer undergoes nonlinear processing in the feed-forward neural network, resulting in more accurate feature values for subsequent outputs. The framework adopts multiple self-attention mechanisms, each computing features at different levels of the image. After computation, the features from different levels are assembled and concatenated, and then input into the feed-forward neural network. The feature values after each layer output undergo layer normalization and residual connections. The data is divided into different samples through layer normalization and then processed in the residual connections to address the problem of gradient vanishing. The goal of each layer is to reduce the occurrence of data loss during transmission and filter out redundant and invalid feature values.

Compared to the U-Net algorithm, the Transformer algorithm demonstrates higher accuracy in processing feature values. However, the Transformer algorithm also has certain drawbacks, such as poor interpretability and limited long-distance learning capabilities. This is due to the attention mechanism’s diminishing ability to capture information as the feature sequence grows, resulting in incomplete feature convergence in large-scale image restoration. To overcome the limitations of both algorithms, some scholars have proposed an improved Transformer algorithm. Experimental results show that this approach not only optimizes the time-consuming training set creation process but also resolves the blurring issue in image restoration when dealing with large-scale image loss.

### Incremental Transformer algorithm

#### Incremental Transformer algorithm principle

This article presents ZITSthat combines convolutional neural networks and Transformer algorithms^45^. The algorithm aims to enhance the generalization ability by improving image preprocessing, optimizing the overall structure to eliminate long-term feature dependencies, and addressing the issue of incomplete image restoration. Image preprocessing plays a crucial role in eliminating irrelevant information and restoring useful real information in the image. The quality and integrity of the output image depend on the processing of information. Different image preprocessing methods primarily aim to better align the original images with the model’s recognition and computation, such as resizing images, image segmentation, and feature recognition. While commonly used restoration model algorithms typically apply simple techniques like cropping and flipping for image preprocessing, in this paper image preprocessing techniques such as bilinear interpolation and mask generation are employed.

Bilinear interpolation is a texture mapping method that performs linear interpolation in two directions to map pixel values. It calculates the weighted average value of the four nearest pixels to the mapping point as the pixel attribute value. Through two rounds of image mapping, the image size changes while preserving the original pixel values, leading to a more realistic image post-processing.

Before applying image input algorithms, standardizing image sizes is an important preparatory task. The purpose of uniform size is to effectively eliminate pixel differences caused by varying image sizes, simplify image processing and algorithmic computation processes, facilitate the input of training sets into algorithms for training, and reduce the burden on computer resources. Van et al. mentioned that 480 × 480 pixel-sized images exhibit unique advantages in the field of image processing^46^. This square size not only helps maintain feature consistency but also preserves sufficient image details while maintaining lower computational complexity. Leveraging the benefits of bilinear interpolation, which preserves frame integrity after image size transformation, this paper utilizes bilinear interpolation to resize images to 480 × 480 dimensions, facilitating subsequent mask processing.

**Figure 15:**
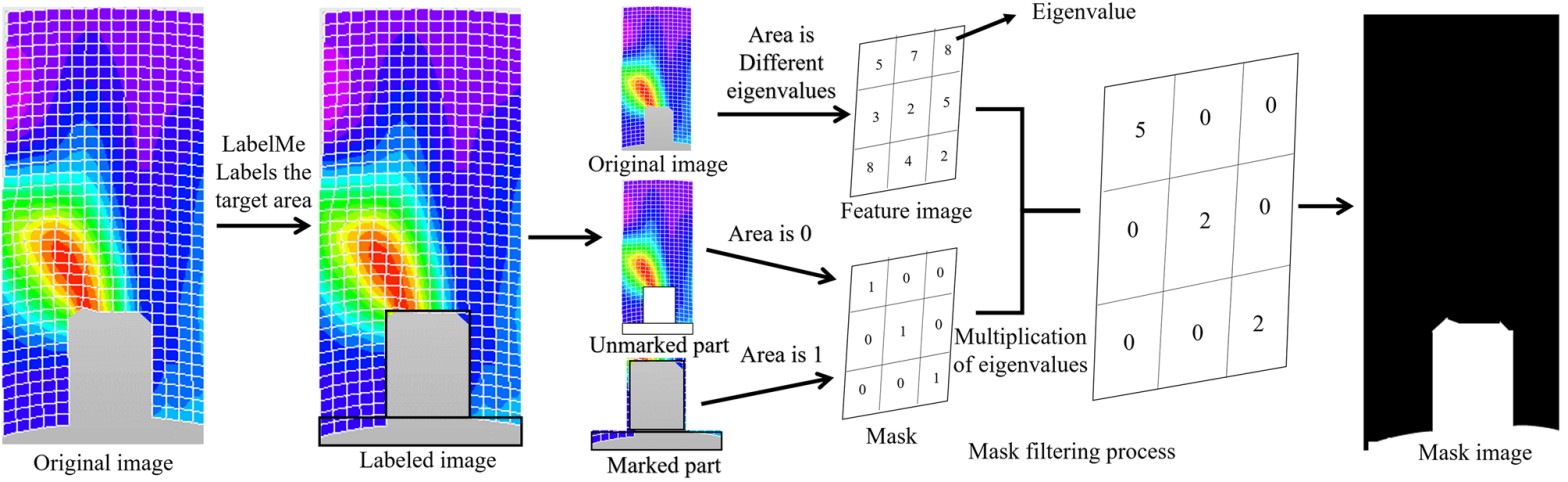
Make mask process diagram.

As shown in Fig. 15, a mask is a single-channel matrix that controls the region of image processing. In the mask, values are divided into zero and non-zero. Non-zero values indicate areas where pixel points from the original image are copied, represented as a white area in the mask. When the mask value is zero, no copying is performed, represented as a black area in the mask. After copying the pixel values into the mask, it is inputted into the model along with the original image. The model processes the masked region while preserving the areas outside the mask. Therefore, the mask specifies the restoration area of the image, significantly improving image processing time and accuracy.

With the advancement of deep learning, significant progress has been made in image restoration. However, restoring images with realistic textures and coherent structures remains a challenging issue. Convolutional neural networks have limited receptive fields and are only able to handle certain regular textures, making them less effective in overall structural restoration, particularly in edge and line recovery. The U-Net algorithm, which is based on convolutional neural networks, has shown great performance in image segmentation. However, it has been observed that there are issues such as texture disappearance and texture discontinuity in the restored images. On the other hand, the Transformer algorithm has addressed these texture-related deficiencies, but it faces challenges in integrating the overall structure of the image.

**Figure 16:**
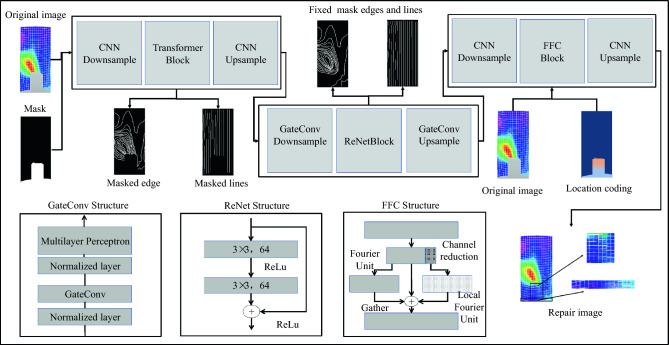
Make Transformer algorithm structure diagram of mask process diagram increment.

This paper introduces an enhanced model based on the Transformer architecture, as shown in Fig. 16. The model comprises three primary components. The first component is the Transformer layer, which processes the image using upsampling and downsampling convolutions. Downsampling reduces the image size and generates thumbnail images^47^, while upsampling enlarges the image to display higher resolution. The second component consists of gate-convolution (GateConv) and Recursive Networks (ReNet). GateConv is an improved network for CNN. Unlike traditional convolutions that treat all input pixels in each channel as valid, GateConv addresses this limitation by adding normalization layers and a multilayer perceptron (a type of feedforward neural network). It employs a *Sigmoid* function for pixel classification, allowing for a learnable dynamic selection mechanism. The *Sigmoid* function is represented as follows:

7 $$Sigmoid = \frac{1}{{\left( {1 + \exp \left( { - x} \right)} \right)}}$$

The ReNet network is employed for target recognition, composed of *ReLU* activation functions and convolutional layers. The activation function plays a crucial role in filtering out unnecessary feature values. The *ReLU* function can be represented by the following Equation^48^:

8 $$f(x) = \left[ {\max (0,x)} \right]$$

Compared to the Transformer algorithm, the second section of this approach eliminates unnecessary feature values. This enables better repair of irregular surfaces, enhances feature highlighting, and ensures a more complete transfer of the required features to the third section.

The third module consists of convolutional layers and Fast Fourier convolution (FFC) layers. The FFC layer is designed to expand the receptive field. It is composed of two interconnected paths: one for partial ordinary convolution and the other for flourier spectral convolution in the spectral domain. These two pathways interact and complement each other by capturing information from different receptive fields. However, the output results of the Transformer algorithm may suffer from resolution loss or partial texture blur. The third module addresses the resolution problem in Transformer algorithm output.

After preprocessing, the original image and the mask are inputted into the model. The algorithm performs feature extraction based on the selected region indicated by the mask. The first module is responsible for the overall structural reconstruction. The output features then pass through the second module for line and edge restoration, and finally, the third module restores textures. The result is the repaired image as the output.

#### Image repair results

This study selected a total of 1213 DIC strain nephograms from various stages of damage as the basic dataset. Two sets of algorithms were used to create different training sets. The U-Net algorithm group requires classification training using labels, so during the creation process, 16 different color strains were labeled using LabelMe software to produce 16 labels. The ZITS algorithm group annotated the missing areas using LabelMe software and then generated masks using Open-Source Computer Vision Library (OpenCV). The NVIDIA Tesla A100 graphics card was chosen as the experimental server, and two sets of experiments were conducted. The U-Net algorithm group took approximately 12 h to train, while the algorithm group in this study took approximately 6 h.

**Figure 17:**
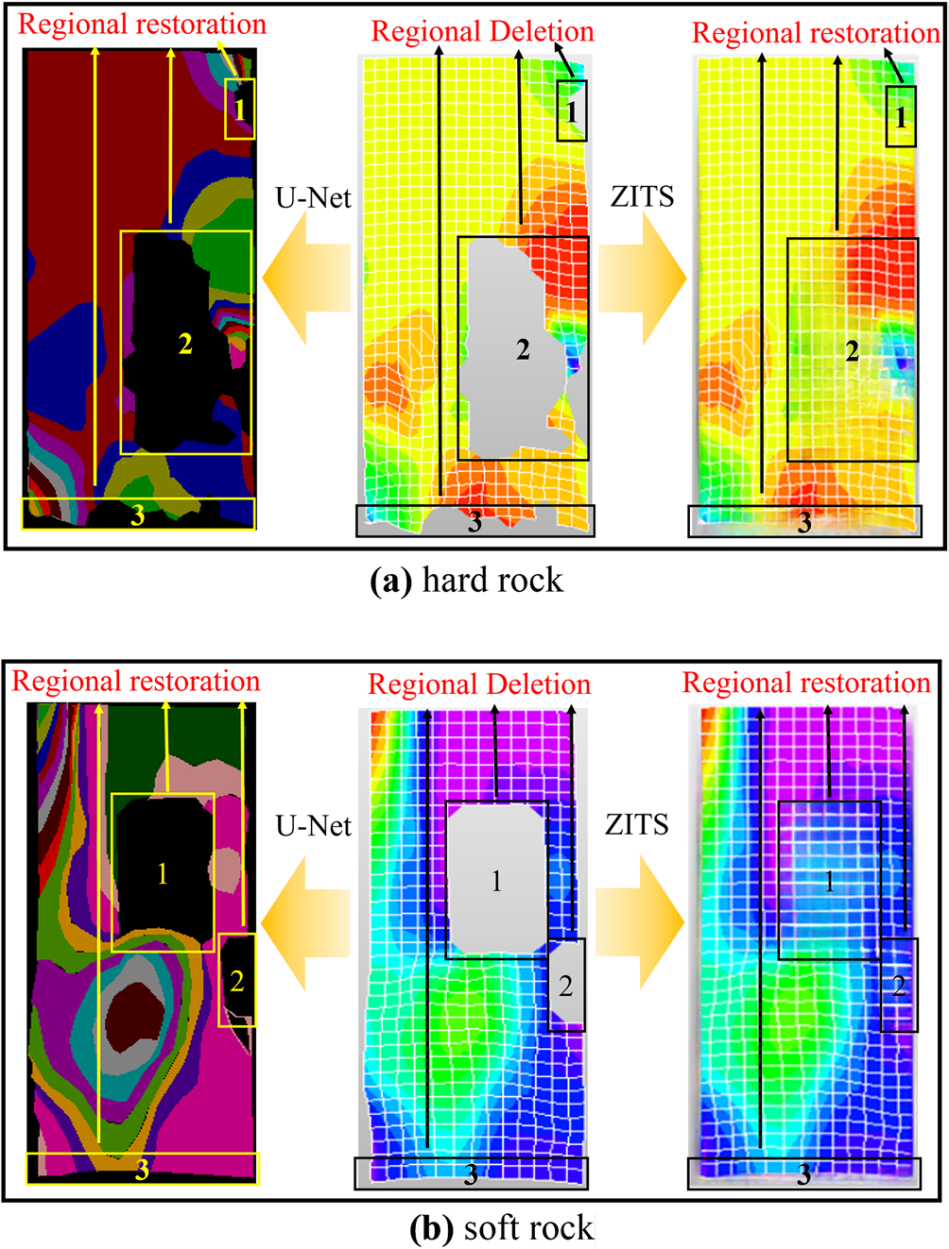
DIC strain nephograms of different rocks are compared with repaired images obtained by two algorithms: (**a**) hard rock, (**b**) soft rock.

During the rock destruction stage after peak load, image defects can occur due to factors such as speckle detachment^49^. Due to space constraints, this article focuses on comparing two models of DIC strain nephogram during the post-peak failure stage. Figure 17 shows that the restored image by the U-Net algorithm does not completely repair the original missing part, resulting in some loss of information. However, the algorithm proposed in this article successfully repairs the missing area in the image without leaving any gaps. Additionally, notable differences are observed in terms of image texture and structure. In terms of image texture, the U-Net algorithm disregarded the division of grids in the original strain nephogram. DIC strain nephograms primarily rely on the displacement changes of monitoring points (i.e., speckle points in Fig. 2) to obtain strain data^7^. The missing grid in the U-Net model affects subsequent experimental observations as it cannot predict the area of lost strain. On the other hand, the algorithm proposed in this paper not only restores the missing region but also maintains the characteristics of the original image and ensures the continuity of the grid in the restored region. This demonstrates the superiority of the Transformer structure in handling complex textures. Regarding the structure, the U-Net algorithm’s restoration image shows misalignment and severe mismatches at the boundaries between the repair and intact areas. In contrast, the algorithm in this article achieves a smooth grid connection between the missing and non-missing areas, with consistent colors at the intersection point of image blocks. Therefore, it can be concluded that the algorithm in this article effectively addresses the discontinuity problem between lines and edges, making it superior to the U-Net algorithm.

**Figure 18:**
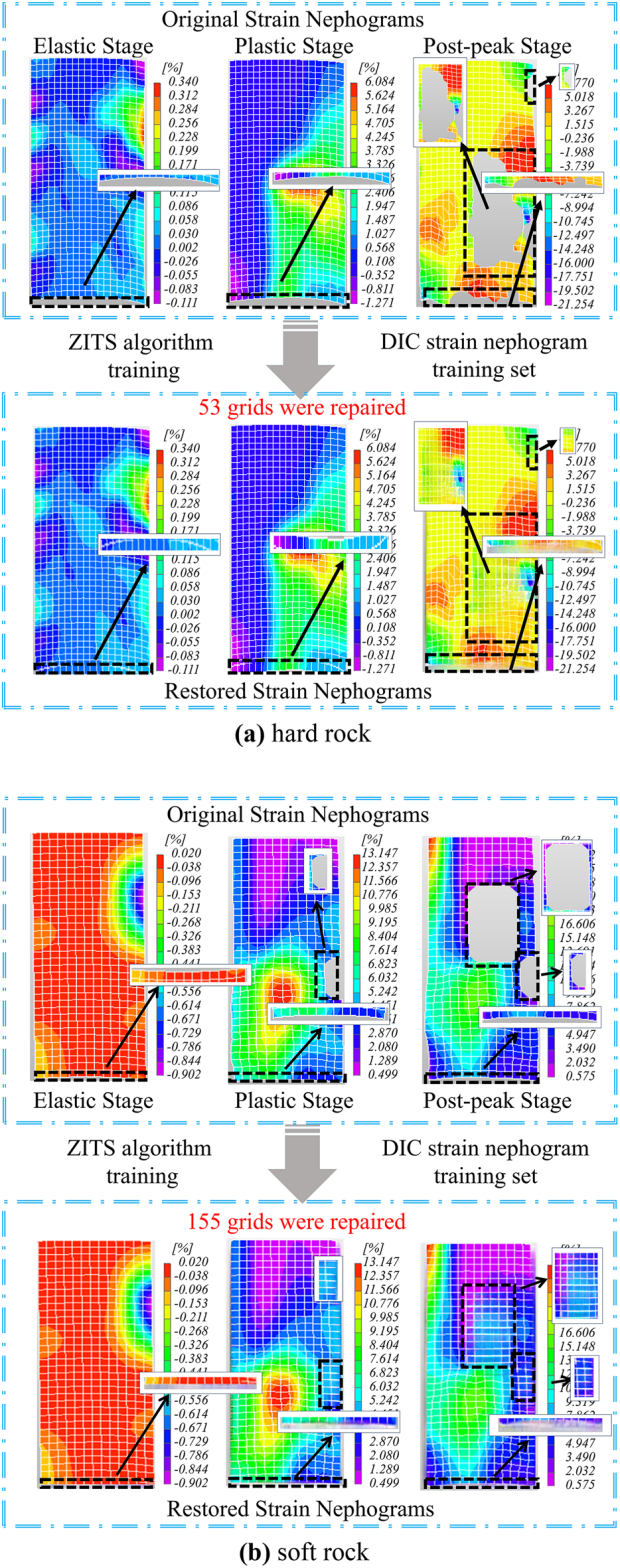
The DIC strain nephograms of different rocks were repaired by ZITS algorithm and compared with the original images: (**a**) hard rock, (**b**) soft rock.

As shown in Fig. 18, the predicted images of the proposed algorithm at different stages of elasticity, plasticity, and post-peak failure are compared with the original images for two types of rocks. It can be observed that each DIC strain nephogram in the hard rock contains 702 grids, while each DIC strain nephogram in the soft rock contains 455 grids. By examining the image pixels, it was found that each grid in the hard rock contains 48 pixels, while each grid in the soft rock contains 132 pixels. During the experimental process, grid loss and dimensionality reduction of displacement data at monitoring points are the main causes of strain data distortion, which mainly occurs at the edges of the images, with a data distortion rate between 8 and 10%.

In Fig. 18a, in the original DIC strain nephograms of hard rock at the stages of elasticity, plasticity, and post-peak failure, there are 702 grids, among which 177 grids are distorted. After being processed by the proposed algorithm, 177 grids and 8496 pixels are repaired, improving the information integrity by 8.41%. In Fig. 18b, in the original DIC strain nephograms of soft rock at the stages of elasticity, plasticity, and post-peak failure, there are 455 grids, among which 129 grids are distorted. After being processed by the proposed algorithm, 129 grids and 17,028 pixels are repaired, improving the information integrity by 9.45%. The repaired DIC strain nephograms provide can provide more complete and accurate global strain data, which provides reliable experimental data support for the establishment of subsequent soft and hard rock constitutive equations.

### Improved incremental transformer algorithm

While using the ZITS algorithm for image restoration of DIC strain nephograms, two issues were identified. Firstly, the quality of image restoration is closely related to the content in the dataset, requiring a sufficient number of DIC strain nephograms to support dataset creation. Although the process of creating masks is faster compared to annotation with LabelMe software, it still poses some obstacles during practical use. Secondly, the ZITS algorithm can only be used on high-performance servers, which are not only expensive but also scarce. Therefore, it is extremely challenging to use the ZITS algorithm for DIC strain nephogram restoration either in field trials or for long-term use in the laboratory. To address these two issues, the following sections will focus on lightweight algorithm processing and the use of suitable datasets to save time and computational resources.

#### Deep separable convolutional networks

In the field of deep learning, most algorithms are designed under the premise of handling complex computations, involving various feature selection processes that increase computational load. Therefore, when utilizing high-performance algorithms to process secondary operations, lightweight processing is employed to reduce the computational complexity and parameters while maintaining system functionality. The purpose is to lower the algorithm’s demands on computational resources, reduce processing time, and investigate the feasibility of deployment on mobile devices. As the DIC strain nephograms requiring restoration in this study do not exhibit complex textures and structures, there are numerous unnecessary computations during the restoration process, leading to prolonged algorithm training time. To address this issue, this study implemented lightweight improvements to the ZITS algorithm by replacing the computationally intense convolutional neural network modules with depthwise separable convolutional network modules.

**Figure 19:**
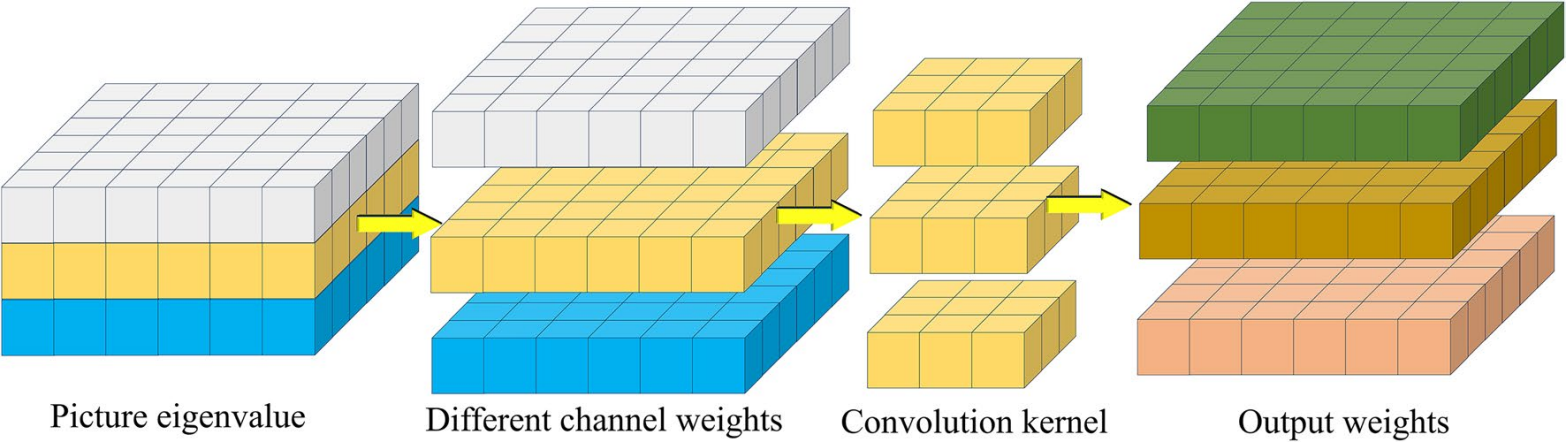
Deep convolutional layer feature processing process diagram.

**Figure 20:**
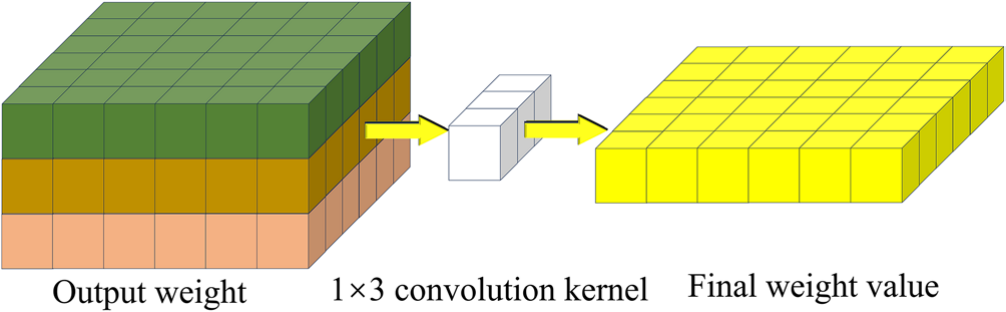
Feature process diagram through point convolution layer.

Depth-wise separable convolutional networks are a variant of convolutional neural networks. They split convolutional layers into depthwise convolutional layers and pointwise convolutional layers, reducing computational and parameter overhead to achieve lightweight designs. As shown in Figs. 19 and 20, the depth-wise convolutional layer applies separate convolutions to the *R* (red), *G* (green), and *B* (blue) channels, generating different weight values for each channel. These values are then processed by the point-wise convolutional layer, which utilizes individual 1 × 3 convolutional kernels to reduce model size and perform channel-wise pooling for dimensionality reduction, effectively reducing parameters and computational overhead. Once the three weight values have passed through the 1 × 3 convolutional kernel, the channel pixel values are fused, resulting in weight values of the same size as a regular convolution. Whereas traditional neural networks consider both channels and regions simultaneously in weight calculations, depth separable convolutions prioritize regions before channels. They individually compute differences across channels, reducing significant computational overhead while cutting some redundant calculations. This approach shortens training time. The following sections will compare the models based on training time and evaluation metrics.

**Figure 21:**
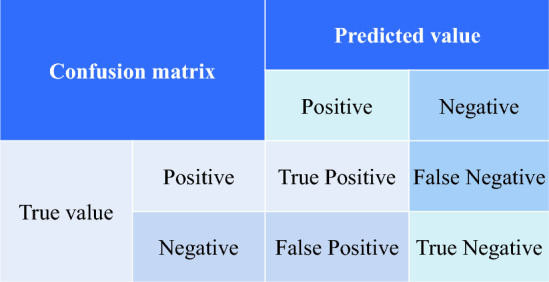
Confusion matrix.

Regarding image accuracy analysis, the ZITS algorithm utilizes three evaluation metrics, *Precision*, *Accuracy*, and *F1-Score*, based on a confusion matrix to assess model training^50,51^. A confusion matrix is an error matrix commonly used for visualizing the performance of deep learning models^52^. As illustrated in Fig. 21, the matrix is a special kind of cross-tabulation consisting of two-dimensional columns representing true and predicted values. Each dimension corresponds to the combinations of classes to be identified, visualized in tabular form, with a decision threshold set between predicted and true values.

The *Precision* metric is calculated based on the confusion matrix and represents the ratio of correctly classified positive samples to the total number of samples classified as positive by the classifier. It is a statistical measure for a subset of samples. The number of samples refers to the actual data points involved in the study or test, describing the features or states of a phenomenon at a specific time point or period, encapsulating specific events with measurement and observation values. The formula for the *Precision* metric is as follows:

9 $$Precision = \frac{TP}{{TP + FP}}$$

where *TP* is the number of correctly classified positive samples; *FP* is the number of samples predicted as positive.

The *Accuracy* metric calculates the accuracy, which represents the proportion of samples correctly detected among all samples. The *Accuracy* metric performs well in classification problems, and the quality of the classifier affects the accuracy. It is used to evaluate the performance of the model. The formula for the *Accuracy* metric is as follows:

10 $$Accuracy = \frac{TP + TN}{{TP + TN + FP + FN}}$$

where *TN* is the number of true negative samples, which are predicted as negative and are actually negative; where *FN* is the number of false negative samples, which are actually positive but mistakenly labeled as negative.

The *F1-score* function is the harmonic mean of *Accuracy* and *Precision* functions, considering both functions equally important in performance evaluation. The overall increase or decrease in the result comes from the combined effects of these two functions, making it a common evaluation metric for deep learning models. The formula for the *F1-score* function is as follows:

11 $$F1 - score = 2 \cdot \frac{Precision \cdot Recall}{{Precision + Recall}}$$

where *Recall* is the recalling rate, indicating the proportion of correctly predicted positive samples out of all actual positive samples. The *Recall* function is shown as follows:

12 $$Recall = \frac{TP}{{(TP + FN)}}$$

The *Loss* function was ultimately chosen as the model completion indicator to evaluate the gap between the model’s predicted values and the actual values. The *Loss* function can determine whether the model can quickly find the optimal solution based on the speed of convergence of the curve. The faster the convergence speed, the faster the model can find the optimal solution. Conversely, a slow convergence speed indicates that the model’s training time is too long, leading to inadequate predictions. In this study, the cross-entropy function was selected, and the formula for the *Loss* function is as follows^53^:

13 $$Loss = - \frac{1}{N}\sum\limits_{i = 1}^{N} {y_{i} \log \mathop {\hat{y}}\limits^{{}}_{i} - \left( {1 - yi} \right)\log \left( {1 - \hat{y}_{i} } \right)} \frac{\delta y}{{\delta x}}$$

where *N* is the number of training samples; *y*_*i*_ is the predicted value, which is the probability of being predicted category; *ŷ*_*i*_ is the label value, which is the probability of being the true category.

#### Dataset optimization

The dataset is a model composed for data visualization, serving as the foundation and core of deep learning algorithms, providing data support for the training and validation set. In the case of repairing graphics with complex textures and structures, the original graphic data is typically used as the dataset. Initially, the most relevant data related to the repair part is extracted from the dataset as the training set, which is then input into the model. The model trains on the corresponding features to identify feature patterns and obtain target weights, resulting in targeted training outcomes. For repairing graphics with simple textures and structures, where feature calculation is simpler, and considering time and cost constraints, the selection of training sets is more diverse.

In most cases, larger datasets can train more stable and higher-performing models. This is primarily because more data’s can provide more information, aiding the model in learning more complex patterns and relationships. However, as the dataset grows to a certain scale, the improvement in model performance begins to diminish, and overfitting may even occur. Therefore, it can be inferred that the number of data points selected for a dataset is not necessarily better when increased but should be evaluated based on the specific task and data conditions. Hence, incorporating diverse features can enhance the model’s generalization capability.

This paper obtained a dataset comprising a total of 1213 DIC strain nephograms from various stages of damage. Careful selection of a portion of the region from each DIC strain nephogram was made to create masks, forming the dataset. This process was time-consuming. To address this issue, the author made changes to the selection of the dataset.

By observing the DIC strain nephogram, it was found that these nephograms can be divided into 16 different colored grid blocks, with each block containing one or more colors. Based on this discovery, the original data was defined using color blocks, and the open-source Places2 dataset was selected. The Places dataset is a commonly used image dataset for visual tasks, and with continuous improvements, the Places1 version has been iterated to the Places2 version, which exhibits superior image categories and quality compared to Places1.The Places2 dataset contains color blocks from various scenes, which can effectively repair the color blocks. By utilizing existing datasets, the need for creating targeted datasets was reduced, thus avoiding significant time wastage. To mitigate potential errors in model training results caused by targeted datasets and open-source datasets, both training sets were deployed on the same server for training, resulting in repair outcomes that could be compared.

**Figure 22:**
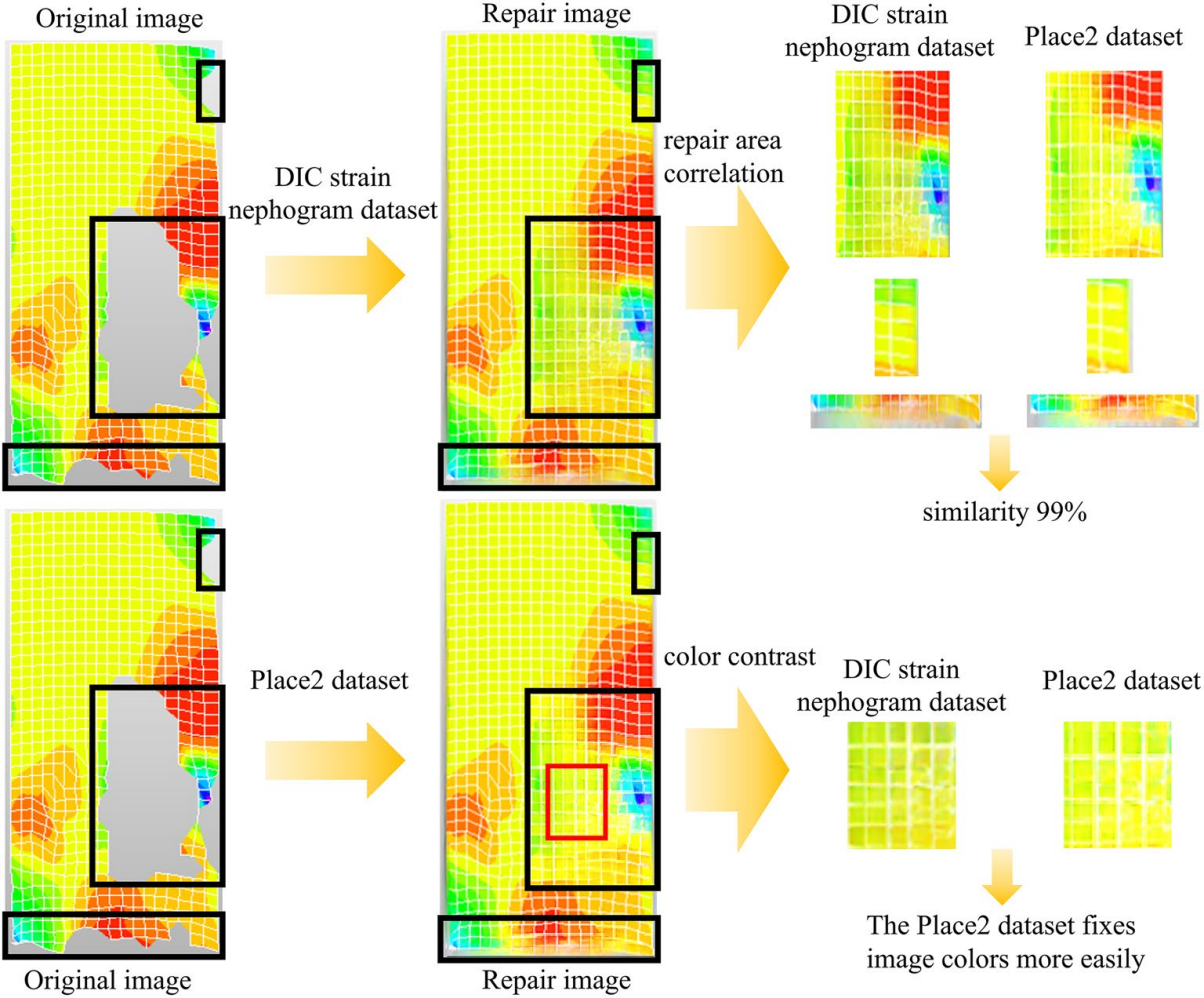
The algorithm is trained by two datasets to repair the result comparison graph.

Comparing the repair images generated by the two training sets, one DIC strain nephogram with high color contrast was selected for illustration, as shown in Fig. 22. Upon observing the images, it was noted that the two repair results were evaluated for errors based on similarity and color vividness. Regarding similarity, by referencing colors, grid curves, and color boundaries, three missing areas in the two repair images were compared, revealing minimal differences between them. In terms of color vividness, a section of the missing area with the same color was selected for comparison, showing that the repair area trained by the Places2 dataset had brighter colors, blending more harmoniously with the unrepaired areas of the original image. This phenomenon was attributed to the imperfect noise handling during the creation of DIC strain nephograms, whereas images in the Places2 dataset underwent more refined technical processing. A comprehensive comparison led to the conclusion that the repair images generated by the Places2 dataset exhibited extremely high similarity to those produced by targeted datasets and excelled in color vividness.

#### Result comparison

To compare the differences between the improved ZITS algorithm and the ZITS algorithm, experiments were conducted using the NVIDIA Tesla A100 graphics card and the NVIDIA GeForce RTX3070Ti as contrasting servers. The Place2 dataset was used as the training set, and both sets of servers were trained simultaneously to compare and analyze the resulting repair images.

**Figure 23:**
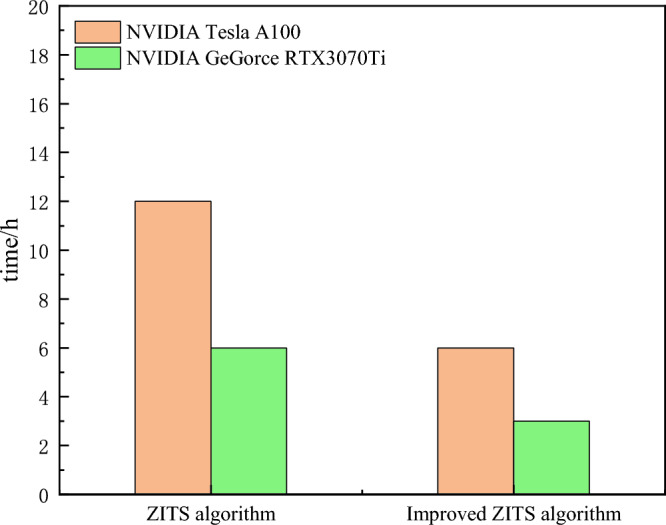
Comparison of training time between two algorithms.

As shown in Fig. 23, the following graph compares the training times of the ZITS algorithm and the improved ZITS algorithm. From the graph, it can be observed that when using the same server, the A100 server group took approximately 6 h for training using the ZITS algorithm, with a result output time of over 3 min. On the other hand, the improved algorithm group took about 3 h for training, with a result output time of 1–3 min, reducing the time by half compared to the ZITS algorithm group. For the 3070ti server group, the ZITS algorithm took around 12 h for training, with a result output time of over 3 min. In contrast, the improved algorithm group took about 6 h for training, with a result output time of 1–3 min, again reducing the time by half compared to the ZITS algorithm group. Based on the available data, it can be concluded that the improved algorithm requires less time for both training and result prediction compared to the ZITS algorithm, thus saving a significant amount of time for image restoration processing on comparable devices.

After training, the two sets of models obtained different evaluation metrics to assess the quality of the repaired images output by the models. The evaluation metrics are shown in Table 2. It can be observed from the table that, due to the reduction in parameter size after algorithm lightweight processing and the removal of some unimportant parameters, the evaluation metrics of the improved ZITS algorithm are slightly lower than those of the original ZITS algorithm.

**Table 2:** Comparison table of evaluation indexes of two algorithms.

Dataset	Algrithm	Edge			Line			Avg
		*Precision*	*Recall*	*F1-score*	*Precision*	*Recall*	*F1-score*	*F1-score*
Places2	ZITS	35.64	27.92	30.39	43.70	60.54	49.35	39.87
Places2	Improved ZITS	35.34	26.28	29.71	43.50	59.48	48.89	39.30

A single image from the validation set typically cannot represent the entire model’s level of restoration. The DIC strain nephogram comprises distinct stages including initial compaction, elastic phase, plastic phase, and post-peak failure stage, each exhibiting unique characteristics. As mentioned earlier, the visual representation of DIC strain nephograms consists of 16 color bands, rendering traditional methods for distinguishing four damage stages ineffective. Algorithms address this issue by deeply training color features to balance performance across different stages.

**Figure 24:**
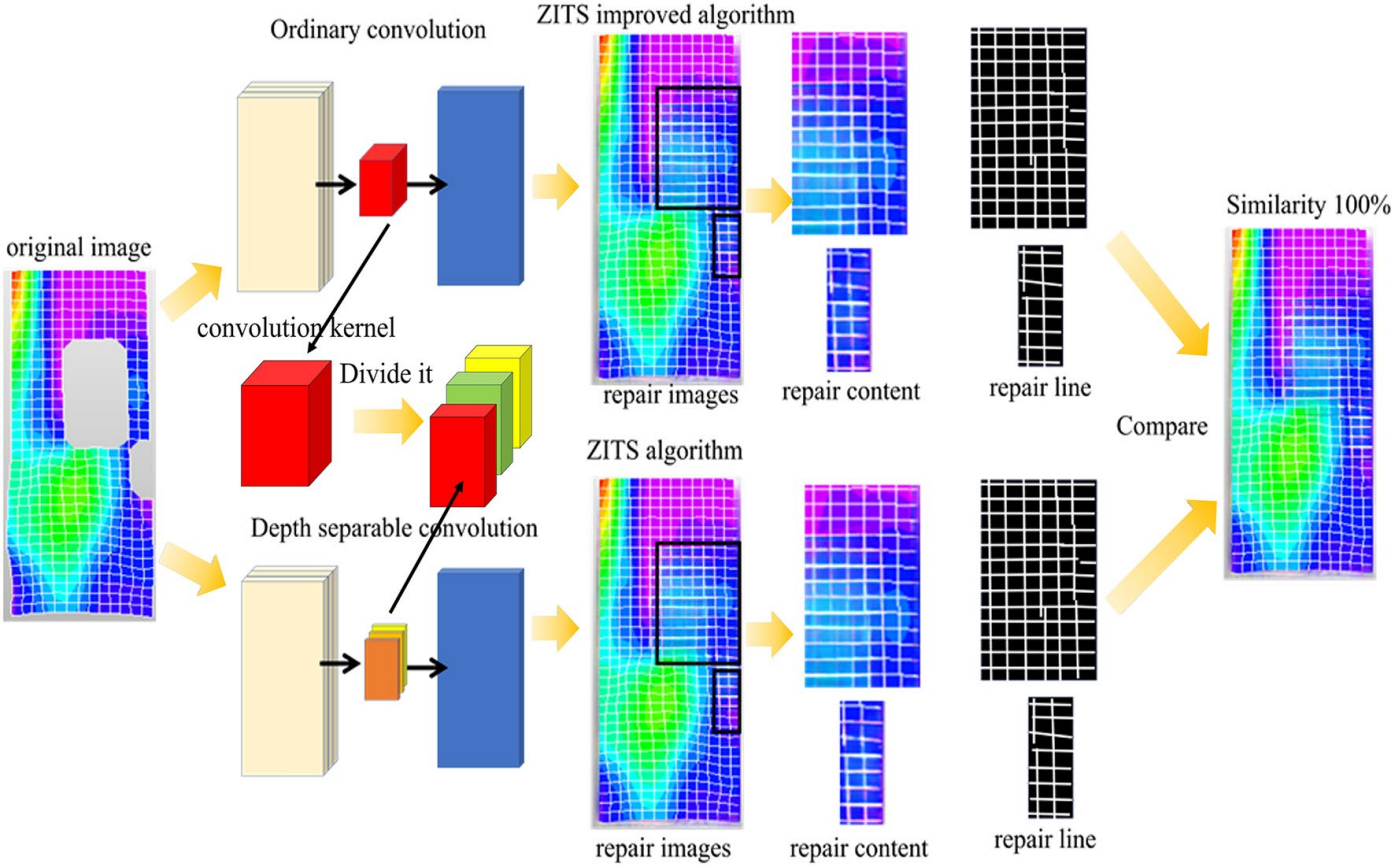
Comparison of repair results between the two algorithms.

Researchers have found that selecting representative images is subjective and non-reproducible^54^. To address this, a two-step approach is proposed. Firstly, features of images in the collection are prominently extracted. Secondly, the selection of representative images is influenced by feature values. To evaluate the disparity in repair image effectiveness between the two algorithms, image quality, clarity of the repaired area, and pre-and post-repair image comparisons serve as assessment criteria. In terms of image quality, DIC strain nephograms rich in strain information with intricate lines are chosen to fully showcase their capability in handling complex details. Regarding the clarity of the restored region, strain nephograms with the largest missing range in the post-peak failure stage are selected as validation samples, ensuring superior repair capability when algorithms repair extensively damaged strain nephograms. Regarding pre-and post-repair image comparisons, images with repair effects at a moderate level are selected. Moderately repaired images to some extent reflect the overall level of the model and its feature advantages. In summary, strains representing the post-peak failure stage of soft rock-like specimens are selected as representative DIC strain nephograms for comparative verification of overall repair effectiveness, as depicted in Fig. 24.

In this study, the repaired images are compared in terms of image content and grid lines. In terms of image content, both algorithms demonstrate a high degree of consistency in colorizing the damaged regions, with no significant differences observed in the color transitions. Further observation of the grid lines reveals that, when isolated and visualized through binarization, the repaired grid sections exhibit smooth connections in some areas and discontinuities in others. However, the disconnected segments are identical in both images, and no additional disconnections are present. Moreover, an analysis of the tilt, quantity, and length of the grid lines indicates a consistent reconstruction of the grid lines. Thus, based on comprehensive comparison, it can be concluded that although the evaluation metrics of the improved ZITS algorithm slightly decrease after training, the content features of the repaired images remain unchanged, and the minor decline in the metrics has minimal impact on the quality of the image restoration results.

## Discussions

### A quantitative damage model

In Chapter 3, this study clarifies the local strain boundaries in different DIC strain nephograms through image data restoration processing, ensuring the accuracy and integrity of the images. As observed in Fig. 18, there are concentrated strain regions, which are densely populated areas of crack development. To assess the rock damage caused by these concentrated strain regions, some researchers propose that all pixels near the micro-crack regions are considered damaged^55^. Therefore, the damage factor is defined as the ratio between the number of pixels in the damaged region caused by cracks and the total number of pixels in the image, and it is defined as follows:

14 $$D_{f} = \frac{{A_{0} }}{A}$$

where *A*_0_ is the number of damaged pixels, where *A* is the total number of pixels.

Due to the potential vulnerability of the areas surrounding cracks, in order to determine the number of pixels damaged due to cracks, it is assumed that the pixels near the damaged pixels are also damaged. The formula is as follows:

15 $$D_{f} { = }\frac{{A_{s} }}{A}$$

where *A*_*s*_ is the total number of pixels in the damaged region and its neighboring grids. The number of pixels can be calculated directly in Matlab based on the DIC strain nephogram combined with the Monte Carlo method, providing experiment-based accurate damage values.

According to the previous research, it is believed that when rocks are subjected to stress, they undergo deformation^56^. However, only when the deformation of rocks reaches a certain degree, damage occur. During the process of rock failure under load, it first goes through the stage of pore compression, where the external pressure is mainly used to densify the rocks, leading to the gradual closure of original elastic structural surfaces, fractures, and pores within the rocks. At this stage, there is almost no stress generated internally in the rocks. Hence, the concept of a damage threshold *ε*_*d*_ is introduced, suggesting that *ε*_*d*_ should be established at a strain of 45% of the peak stress of the rock. The distribution of internal rock damage is determined by comparing the maximum strain value *ε*_*max*_ to *ε*_*d*_, where *ε*_*max*_ > *ε*_*d*_ indicates regions of damage within the rock. Therefore, in the DIC strain nephogram, the strain region where *ε*_*max*_ > *ε*_*d*_ is identified as the damaged area. In summary, the damage factor is defined as follows:

16 $$D_{f} = \left\{ {\begin{array}{*{20}l} 0 \hfill & {\left( {{0} < \varepsilon \le \varepsilon_{d} } \right)} \hfill \\ {\frac{{A_{s} }}{A}} \hfill & {\left( {\varepsilon > \varepsilon_{d} } \right)} \hfill \\ \end{array} } \right.$$

When *D*_*f*_ = 0, it represents undamaged soft or hard rocks, when *D*_*f*_ = 1 represents completely damaged soft or hard rocks. It is important to note that Eq. (16) differs from the elastic stage model during the initial densification stage and uniaxial compression of rocks. Therefore, when inverting the stress–strain curve, the strain termination point *ε*_*d*_ for the densification stage should be the starting point of the elastic stage in the rocks.

Based on the above analysis, the model shown in Eq. (16) is highly dependent on the accuracy of the DIC strain nephogram. If the image has issues like missing data or blurring, it will have a significant impact on the determination of damage values. However, in this study, the algorithm proposed in this paper for the correction and prediction of DIC strain nephograms effectively addresses these issues. By establishing strain thresholds, the characteristics of undamaged changes in the initial densification stage are compensated for, and a segmented curve damage model applicable to both the initial densification stage and subsequent damage characteristics is established.

### Model verification

**Figure 25:**
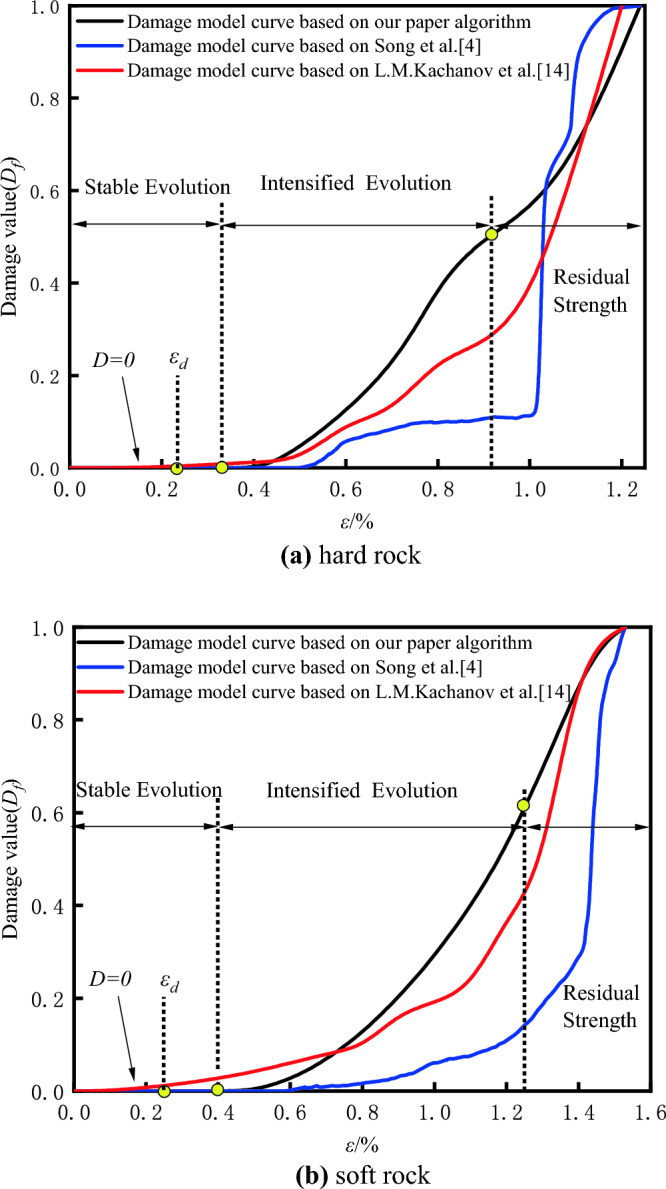
Comparison of damage strain curves of different rocks based on three damage constitutive models: (**a)** hard rock, (**b**) soft rock.

To validate the reliability of the quantitative damage models for soft and hard rocks, as well as the rationality of the data optimization effects of the algorithm proposed in this paper, simulation analyses were conducted on both soft and hard rocks. The improved damage quantitative model established by Eq. (16) with the DIC-based damage analysis model mentioned by Song and the AE-based model mentioned by L.M^4,14^. The fitted damage strain curves are shown in Fig. 25 for comparison.

Research by Gong and colleagues indicates that damage variable growth exhibits three distinct phases^57^: the stable damage evolution stage, the intensified damage evolution stage, and the post-peak residual strength stage. These correspond to the consolidation phase, elastic–plastic phase, and post-failure phase of the stress–strain curve, respectively. During the consolidation phase, internal specimen porosity is reduced to closure, resulting in zero damage to the rock. As the material enters the elastic–plastic phase, internal micro-crack expansion accelerates, and new cracks continually extend, causing a rapid increase in the damage rate. During the failure stage, the specimens’ overall strength has already been compromised after the peak strength, the damage growth rate starts to stabilize, and finally, approaching 1 signifies that the rock has been completely fractured.

According to Fig. 25, the damage strain curve obtained from the DIC-based damage analysis model in soft rock specimens exhibits a concave shape. The rate of damage growth gradually increases until the final destruction stage, indicating an overall trend of accelerated growth, which contradicts the typical damage pattern observed during rock failure. In addition, rocks experience significant damage only in the later stages of the plastic phase, which also contradicts the expected physical changes during the rock loading process. Therefore, it is evident that the damage analysis curve derived from this method lacks rationality. On the other hand, the damage model curve based on acoustic emission technology shows a slow ascent during the elastic stage, compensating for the damage variation in this stage. It also addresses the issue of the plastic stage curve accelerating too quickly to reach 1. However, in soft rocks, damage begins to occur from the very start of the loading and compaction stage, which is not in line with normal physical changes. This discrepancy is likely due to a large amount of noise generated during the test affecting AE signals and leading to misinterpretation, thus impacting the results of the experiment. Upon reviewing the damage analysis curve based on repaired DIC images and optimized through the algorithm presented in this paper in comparison with the other two curves, it is found that the damage curve from this model is smoother, with the rate of damage increase following a “slow-rise-then-decline” trend. Additionally, two distinct inflection points can be identified during the elastic and failure stages. This indicates that during the elastic phase, as the rock pores close, the external load is directly applied to the rock matrix, initiating the expansion of cracks, and leading to a sudden damage increase. In the post-peak failure stage, the overall strength of the rock has largely been lost, leaving only residual strength to counteract the loading effect, causing the damage growth rate to decrease and the curve to flatten. Therefore, the damage model optimized using the algorithm in this paper aligns more accurately with the actual damage evolution observed.

By assuming isotropic internal damage of the rock and applying the principle of equivalent strain, the material’s relationship among strain, damage, and stress under uniaxial loading can be quantitatively determined^58^:

17 $$\sigma = E\varepsilon \left( {1 - D} \right)$$

where *E* is the Young’s modulus of the rock, expressed in MPa.

By integrating Eq. (17) with Eq. (16), we can derive the stress–strain relationship obtained from the damage model optimized by the algorithm presented in this paper:

18 $$\sigma = \left\{ {\begin{array}{*{20}l} 0 \hfill & {\left( {{0} < \varepsilon \le \varepsilon_{d} } \right)} \hfill \\ {E\varepsilon \left( {1 - \frac{{A_{s} }}{A}} \right)} \hfill & {\left( {\varepsilon > \varepsilon_{d} } \right)} \hfill \\ \end{array} } \right.$$

**Figure 26:**
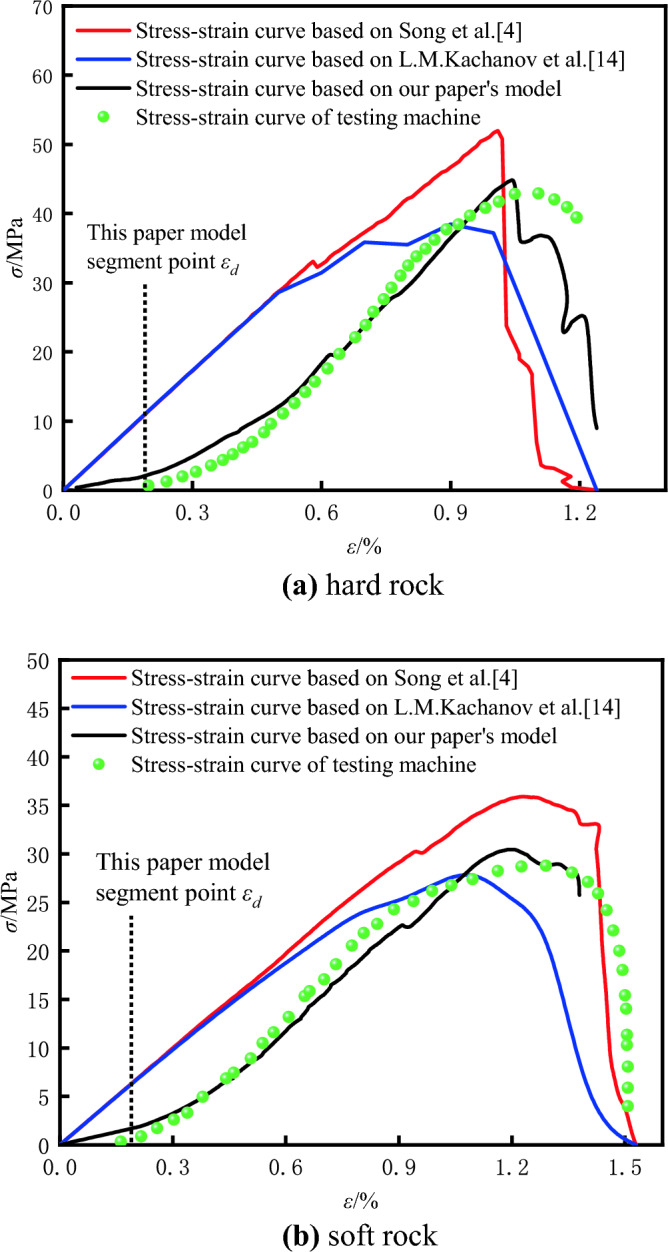
The stress–strain curves of different rocks are compared based on three damage constitutive models and testing machine: (**a**) hard rock, (**b**) soft rock.

It is particularly noteworthy that Eq. (18) differs from the model in the initial consolidation and elastic phases during the uniaxial compression of rocks. Consequently, when inverting the stress–strain curve, the endpoint of the strain in the consolidation phase should correspond to the starting point of the rock’s elastic phase. Based on this, we can derive the stress–strain curve informed by the optimized damage model, as illustrated in Fig. 26.

After optimizing the damage model algorithm in this paper, and comparing it to the stress–strain curves obtained from the damage models mentioned by the DIC-based damage model, as well as those from the AE-based damage model, and the original data curves, it is evident that the optimized model aligns more closely with the curves derived from the original data. The strain data derived from DIC experience missing values due to factors like speckle dropout during post-peak failure stages, which hinders an accurate representation of damage evolution at this phase. Consequently, the back-calculated curves show a significant drop, inconsistent with the rock uniaxial compression process. Although AE data do not suffer from image loss issues, noise during the experimental procedure results in curve deviations, making the response to damage evolution at various stages less reliable. On observing the stress–strain curves based on AE technology, a substantial divergence is found from the stress–strain curves recorded by the testing machine. During the rock compaction stage, internal pores gradually close, and almost no damage occurs. However, environmental noise causes some signals to exceed the threshold, leading to errors in damage analysis. By observing the curves analyzed using the damage model of DIC images repaired by the optimized algorithm proposed in this paper, the initiation point of damage should correspond to the start of the rock’s plastic phase. During the compaction and elastic stages, rock damage is virtually zero; during the plastic phase, accelerated damage due to stress leads to a sudden increase in damage, with corresponding stress rapidly climbing to peak strength. In the post-peak failure stage, as rock strength decreases, the damage growth slows down, corresponding to a sharp drop in stress, and the stress–strain curve plummets quickly. The stress–strain curves derived from this paper’s model reach an agreement of over 90% with the experimentally obtained curves, indicating that our model more accurately reflects the trend of specimen damage evolution, thus validating the model’s rationality.

## Conclusions

In response to the issue of partial distortion in traditional DIC technology images, this paper focuses on both soft and hard rocks as research subjects. By employing the improved ZITS algorithm for the identification, correction, and prediction of DIC images, the following conclusions have been drawn:

1. When constructing damage constitutive models using strain maps generated by DIC technology, data loss due to speckle fragmentation and detachment leads to significant errors in damage analysis. Concurrently, as the stress–strain curves inferred from the damage strain curves exhibit substantial discrepancies compared to those obtained from the testing machine, repairing DIC strain nephograms becomes a crucial step to enhance the effectiveness of rock damage analysis based on DIC technology. The improved DIC strain nephograms have shown an 8% to 10% increase in information completeness, and the enhanced damage constitutive models proposed using the restored image data result in inferred stress–strain curves that align more closely with those from the testing machine. This demonstrates that the repaired DIC strain nephograms effectively improve the precision of damage analysis.
2. The operation of the ZITS algorithm not only demands high server performance but also involves a complex and time-consuming computational process. In this study, lightweight measures are adopted to mitigate these challenges. By replacing the traditional convolutional network with a depthwise separable network, the new ZITS algorithm allows for parallel computation of multiple features, reducing both computational workload and parameter count. The improved ZITS algorithm not only cuts the computation time by approximately half compared to its predecessor but it can also be used on standard low-configuration servers. Additionally, the enhanced ZITS algorithm has no negative impact on image detail. Therefore, adopting the modified ZITS algorithm proposed in this paper will significantly save time and money in the field of DIC image restoration and improve the efficiency of analyzing rock damage using DIC technology.

## Data Availability

The datasets generated during and/or analysed during the current study are available from the corresponding author on reasonable request.
